# Multiomics Research Strategies in Cancer: A Growing and Innovative Field

**DOI:** 10.1002/mco2.70644

**Published:** 2026-03-24

**Authors:** Zhenhua Du, Xiaomei Liu, Zhi Lv, Bengang Wang, Yu Xia, Wala Abduljabbar Mohammed Al‐Duais, Lirong Yan, Fuqiang Zhang, Yanke Li

**Affiliations:** ^1^ Department of Gynaecology and Obstetrics Shengjing Hospital of China Medical University Shenyang China; ^2^ Department of Anorectal Surgery First Hospital of China Medical University Shenyang China; ^3^ Department of Hepatobiliary Surgery First Hospital of China Medical University Shenyang China; ^4^ The First Laboratory of Cancer Institute First Hospital of China Medical University Shenyang China

**Keywords:** biomarkers, cancer, deep learning, multiomics, precision medicine, single‐cell omics, spatial omics

## Abstract

Cancer is a highly complex and heterogeneous disease involving multiple pathophysiological events. Despite significant advances in modern medicine, the molecular mechanisms of cancer are still largely unknown. Omics methods have opened new avenues for identifying cancer biomarkers and elucidating disease pathogenesis. However, single‐omics approaches only provide a limited understanding of biological mechanisms. The comprehensive analysis of multiomics data will provide useful insights for the pathogenesis, identification of therapeutic targets, and discovery of biomarkers in cancer. Here, we reviewed the disease signatures of cancer. We then reviewed the current state of multiomics biomarkers research in cancer. To further delineate the upstream pathogenic changes and downstream molecular effects of cancer, we also discuss the current strategies for integrating multiomics data using deep learning approaches. In addition, single‐cell and spatial omics are being used to guide treatment strategies, risk assessment, and early diagnosis, as well as their potential impact on precision medicine. Despite the relative youth of the field, the development of single‐cell and spatial omics promises to provide a powerful tool for elucidating the pathogenesis of cancer.

## Graphical abstract

This review highlights multiomics strategies in cancer research, focusing on integration methods from genomics to microbiomics. Using colorectal cancer as a key example, it discusses biomarker discovery, data integration via deep learning, and the roles of single‐cell and spatial omics. The article underscores the translational potential of multiomics in advancing precision oncology and improving clinical outcomes.

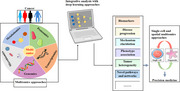

## 1 Introduction

Cancer is a multifaceted disease. It can strike at any age, develop slowly or rapidly, be benign or malignant, and show up in nearly any cell, tissue, or organ. There are now about 200 distinct cancer kinds known to exist, and the number is constantly increasing. The pathology of cancer is a very complex pathological process involving a variety of pathophysiological events. A better understanding of the pathophysiology of cancer will enhance preventive, diagnostic, and therapeutic strategies. The identification and monitoring of molecular biomarkers are favorable for the diagnosis, prognosis, and therapy strategies of disease [1]. However, certain drug trials have had disappointing outcomes, suggesting that the antitumor effects in people with cancer are more complex than in the animals used in the experiments. Therefore, it is essential to create new approaches to cancer diagnosis and treatment.

**FIGURE 1:**
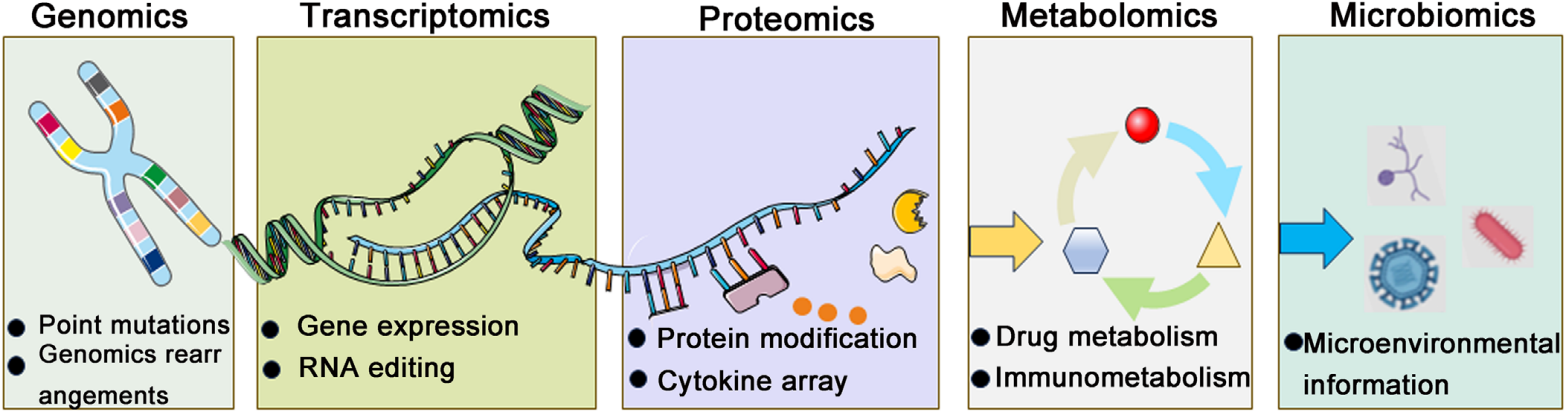
Schematic representation of multiomics approaches (by FigDraw). Multiomics approaches provide comprehensive knowledge about cellular components and biomolecules, including genes, proteins and metabolites, among others.

Omics was extensively used in the last decades to find biomarkers for a variety of diseases [2]. The advancement of medicine has been significantly hastened by advances in omics [3]. To track relevant information at various biological phases, different methods of omics were employed, such as genomics, transcriptomics, proteomics, metabolomics, and microbiome [4] (Figure 1). Comprehensive information on cellular constituents and biomolecules, such as genes, ribonucleic acid (RNA), proteins, and metabolites, has been made possible by omics approaches. Combining the proteomic signature of cancer with patient prognostic assessment is a useful tool for discovering new biomarkers, identifying drug targets, and assessing cellular responses to external stimuli. Multiomics is increasingly yielding promising results, and these approaches are complementary, especially if different multiomics methods are applied to the same patient. Multiple types of omics data are used to obtain a comprehensive understanding of cancer and to develop precise drug therapies.

Multiomics approaches have been creatively used in cancer research, especially in the evaluation of tumor heterogeneity and T‐cell infiltration [5]. By providing genomic, transcriptomic, protein, and metabolomic data, multiomics technology can provide more in‐depth understandings of cellular properties and regulatory mechanisms. Furthermore, the application of single‐cell and spatially multiomics techniques is helping us to understand the spatial variability of disease molecules and their molecular origins [6]. However, there are still a number of limitations on the application of multiomics technology to cancer treatment. Further basic research is needed to ensure the accuracy and reliability of these techniques. Technical dependability, standardized data transmission protocols, and other challenges like data analysis optimization are only a few of the numerous challenges that need to be addressed. The resolution of these issues and the reinforcement of pertinent study findings will come from the ongoing development and improvement of associated technologies. Our comprehension of cancer will undoubtedly improve with the use of multiomics techniques.

In this paper, we first look at the multiomics approaches. Colorectal cancer (CRC) is the third most common cancer worldwide, with a prevalence rate of 10% among all cancers [7, 8]. Then, we reviewed the current state of multiomics research in CRC as examples. In addition, we also reviewed recent applications of deep learning (DL) methods to integrate multiomics data, which aids in the characterization of pathogenic alterations and molecular influences in cancer. Subsequently, we discussed the role of multiomics research in personalized medicine. Furthermore, the application of single‐cell and spatial multiomics is influencing precision medicine by directing treatment strategies and risk evaluation. It is clear that the field of multiomics is moving toward single‐cell and spatial analysis, which is certainly one of the most powerful tools at our disposal at the moment. This will allow us to perform a more detailed and specific analysis of cancer.

This section will analyze the principles and progress of multiomics approaches in four dimensions: genomics, transcriptomics, proteomics, and metabolomics, with a focus on the application of integrated analysis of different omics in disease research.

## 2 The Multiomics Toolbox: Core Technologies and Analytical Dimensions

The conventional method of finding new drugs is dangerous, expensive, and time consuming [9]. With higher sensitivity and resolution than traditional research methods, omics offers solutions for drug target discovery, molecular diagnostics and prognosis [10, 11]. As a novel and developing molecular tool, omics has advanced significantly over the last few decades and is now a vital component of biological and medical research. Omics techniques are essential for fully comprehending biomolecules and cellular constituents, including genes, proteins, and metabolites [12].

### 2.1 Genomics and Epigenomics

Scientists completed and discovered the sequence of the human genome in 2001, greatly increasing the ability to find and identify genes responsible for inherited diseases [13]. There is no doubt that the Human Genome Project is the driving force behind genomics. The structure, function, and interactions of an individual genome are studied in genomics [14]. Thousands of genomic sequences have been compared and analyzed thus far [15]. Many studies only look at cell populations, and researchers only get the average genetic information from cell populations, while the information of cell subsets is often overlooked [16]. The influence of cellular heterogeneity on gene expression is difficult to identify by traditional methods. Single‐cell sequencing has greatly advanced when compared with conventional methods, due to the rapid development of sequencing techniques [17]. Both changes in genome structure and nucleotide differences inside individual cells are detected using single‐cell sequencing techniques [18]. In single‐cell studies, whole‐genome and whole‐exome sequencing have been generally utilized [19]. However, the absence of a comprehensive genome‐wide amplification technique and data description still results in some restrictions [20]. Consequently, merging datasets from many platforms and integrating disparate information sources yields more trustworthy information [21].

Through chemical alterations in proteins and nucleotides, epigenetic modifications can impact gene expression and function without changing the nucleotide sequence itself. An increasing amount of evidence suggests that the genesis and spread of human malignancies are significantly influenced by epigenetic changes, which primarily impact chromatin accessibility and epigenomic modifications [22]. Histone alterations, which make up the biggest category of analyzed chromatin marks, are crucial for the development and maintenance of malignant cell phenotypes in a variety of cancer types. One often used technique for profiling histone changes is chromatin immunoprecipitation sequencing, or ChIP‐seq [23]. Among RNA modifications, the most common internal RNA modification in eukaryotes is N^6^‐methyladenosine (m^6^A). Many high‐throughput sequencing methods can be used to detect m^6^A, such as m^6^A/MeRIP‐seq [24], m^6^A‐REF‐seq [25], m^6^A‐Seal [26], and so on. One of the most extensively researched aspects of the eukaryotic genome is chromatin accessibility, which controls physical access to deoxyribonucleic acid (DNA) and is crucial for creating and preserving cellular identity [27]. Numerous epigenomic characteristics that are implicated in tumor start, development, metastasis, and immune evasion have been thoroughly characterized in cancer patients by pursuing these epigenetic techniques [28]. Clinical practice is beginning to use a variety of epigenetic biomarkers and treatment approaches [29].

### 2.2 Transcriptomics and Epitranscriptomics

Transcriptomic analysis provides characterizing information about the RNA species produced within cells. The classification of all transcript species and measurement of the variations in each transcript expression levels during development and under various circumstances are the goals of transcriptomics [30]. Because of the quick advancement of next‐generation sequencing techniques and DNA microarray technologies, transcriptome analysis is now widely used [31]. Despite having almost similar genomes, every organ and tissue in humans has a distinct gene expression profile [32]. A deeper comprehension of cellular function and disease causation is facilitated by comparing several transcriptome data types from healthy and sick animals. It is expected that transcriptomics would be utilized in clinical diagnostics as it makes it possible to identify certain gene expressions linked to disease. Similarly, single‐cell transcriptome sequencing has demonstrated clear advantages in evaluating the diversity of individual cells and the intracellular transcriptional environment [33]. However, low RNA quantity and low reverse transcription and amplification efficiency limit the investigation of single‐cell transcriptomes [34]. These limitations are removed by microchip‐based methods, which enable the processing of trace quantities of RNA in nanoliter processes [35].

Epitranscriptomics is the study of posttranscriptional modifications (PTMs) in RNA molecules that affect the fate of RNA without altering its sequence [36, 37]. To date, more than 170 different kinds of RNA modifications have been identified in various RNA species, such as noncoding RNA, messenger RNA, transfer RNA, and ribosomal RNA [38]. The most common alteration in eukaryotic mRNAs and a common feature of several ncRNA types is m^6^A [39, 40]. Another epigenetic layer that is essential for controlling chromatin state and gene transcription is the epigenome, which is made up of DNA and histone modifications. The most prevalent DNA alteration in animals is DNA methylation, which mostly forms 5‐methylcytosine (5mC) [41]. The TET family proteins mediate the removal of 5mC by an oxidation process, whereas DNMT1, DNMT3A, and DNMT3B accelerate its installation. Histone is essential for the structure of chromatin and undergoes a number of chemical modifications, mostly on the flexible N‐terminal tail extending from the nucleosome. Types of histone modifications include phosphorylation by histone methyltransferases and phosphatases, ubiquitination by ubiquitin ligases and deubiquitinating enzymes, and acetylation controlled by histone acetyltransferases and histone deacetylases, among others [42].

### 2.3 Proteomics and PTMs

Proteomics is the large‐scale and high‐throughput study of the entire proteome of an organism or system, especially the structure and function [43]. Proteomics is a useful tool for comprehensive understanding of genome expression and further a supplement to genome translation and modification studies. These fast‐evolving proteomics approaches have been used in clinical stroke research to better understand the biology of the disease and thereby identify many potential biomarkers. Nowadays, two‐dimensional gel electrophoresis has been a well‐established approach for studying proteomics. However, how to enhance the capacity, sensitivity, resolution, and detection accuracy of two‐dimensional gel electrophoresis is the key issue. Presently, liquid chromatography–mass spectrometry (MS) [44], gel electrophoresis–liquid chromatography–MS [45], capillary electrophoresis–MS [46], and other chromatographic techniques are increasingly used in proteomics because of their high resolution and sensitivity [47]. MS is an important approach in proteomics analysis for detecting and quantifying massive proteins and peptides in a single sample [48]. In addition to these techniques, whole‐proteome microarrays are commonly used to determine target antigens, evaluate immune responses and biomarkers, and for clinical diagnosis [49].

Furthermore, multiple PTMs that can swiftly and reversibly react to intracellular or environmental stimuli enhance the extraordinary variety of proteins, which will ultimately prove to be a useful resource for biomarker candidates [50]. Today, MS‐based proteomics provides a global picture of proteome diversity under a particular state, such illness, by enabling the simultaneous analysis of thousands of proteins and their expressional alterations [51]. PTMs, which are chemical changes on certain amino acids, alter the conformation, activity, interaction, stability, and spatial distribution of the majority of eukaryotic proteins [6]. As a result, they work as quick and reversible switches and carry out regulatory tasks in a very “cost‐effective” manner. The fact that phosphorylation is a key regulator of the cell cycle, proliferation, apoptosis, and signal transduction pathways serves as an example of this. A growing body of research indicates that a number of human disorders are linked to aberrant PTMs occurrences. It has been demonstrated that PTMs like phosphorylation, glycosylation, acetylation, ubiquitination, methylation, and citrullination contribute to the development, spread, and metastasis of cancer. To identify targets with biological and therapeutic potential, PTMs must be globally profiled.

The majority of PTMs in proteomes cannot be effectively identified or quantitatively characterized without previous enrichment due to their low abundance and substoichiometry. Covalent tagging and noncovalent interactions between the PTMs of interest and its selective probes are the two main types of enrichment approaches utilized in global PTMs profiling procedures, depending on the chemistry involved. For every kind of PTMs, distinct enrichment techniques based on one of the two chemical kinds have been created. The enriched PTMs were then put through a proteomic analysis, which uses MS to determine the mass characteristics of the daughter ion and the particular mass shift of the parent ion.

### 2.4 Metabolomics

Metabolomics is the qualitative and quantitative analysis of multifactorial, dynamic, and metabolic responses to pathological and physiological stimuli in living systems [52]. The approach of metabolomics, as a new member of the omics research community, was first proposed in 1999 by Nicholson et al. [52]. Small compounds created, destroyed, and transformed within cells are separated using chromatography methods such as gas chromatography, liquid chromatography, and capillary electrophoresis [53]. The most widely utilized downstream methods for identifying metabolites are nuclear magnetic resonance (NMR) and MS. NMR or MS‐based metabolomics methods are used to identify biological samples, which are then combined with multivariate statistical analysis to discover compounds with high specificity and sensitivity as potential disease biomarkers. Additionally, the data were combined with the data from other omics approaches (genomics, transcriptomics, and proteomics) to identify the molecular basis of metabolite alterations, which was followed by molecular biology methodologies to reveal the disease's underlying pathogenesis. Two common pattern recognition methods and statistical tools for analysis of NMR and MS data are principal component analysis and partial least squares [54]. Other metabolomics programs for MS analysis include XCMS [55], MZmine [56], MetAlign [57], and MathDAMP [58].

### 2.5 Microbiomics

A new area of omics technology called microbiomics studies the symbiotic or pathogenic relationships that exist between microbial populations. It has the unique ability to provide a telescopic panoramic view of the whole community dynamics, as well as a microscopic view of the behavior of individual genes, proteins, or metabolites in a large population [59]. The human microbiome is a complex multikingdom community that interacts with the host in a symbiotic manner at several bodily locations. A range of multifactorial illness disorders and other physiological systems are impacted by host–microbiome interactions. Over the last 10 years, it has been proposed that microbiome populations affect the onset, spread, metastasis, and responsiveness to therapy of a variety of cancer types. Improved molecular knowledge of such cancer‐modulating interactions and consequences on cancer therapy are thought to be of considerable scientific value and therapeutic relevance, even though the causal evidence of microbial impacts on cancer biology is still being uncovered.

**FIGURE 2:**
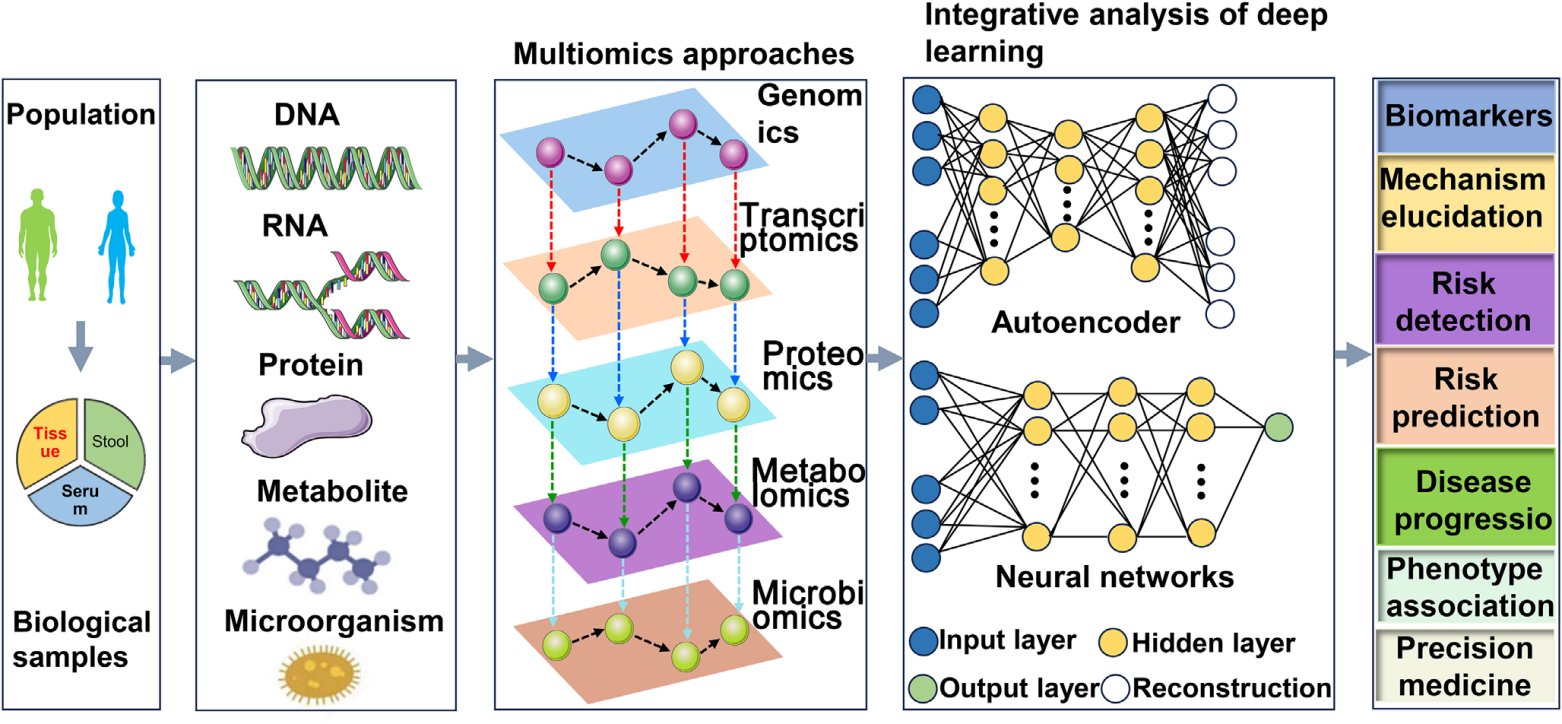
Schematic representation of different omics approaches describing the layout of human cancer (by FigDraw). It illustrates how deep learning methods may be applied at the multiomics level and describes how integrating multiomics may help in cancer treatment.

Current research is using multiomics approaches to identify biomarkers for treatment effectiveness and prognosis in cancer. Nonetheless, the number of studies applying multiomics analysis methods is still limited. Integrating multiomics data with mathematical models helps to elucidate the biological processes of cancer. A thorough examination of cellular functions may be essential to comprehending the pathophysiology of cancer. Combining multiomics approaches may make it possible to find significant pathways. Multiomics is a cutting‐edge technique that allows for the simultaneous and in‐depth analysis of multiple molecular compartments and their changes. By facilitating integrated analysis for a better understanding of how molecules interact and, as a result, improving the prognosis of diseases, the resulting information has completely changed biology and precision medicine (Figure 2).

## 3 Strategies for Multiomics Data Integration: From Statistics to DL

This section examines the computational frameworks for integrating heterogeneous multiomics data. It begins by outlining key challenges such as data dimensionality, noise, and biological variability, then systematically reviews early, intermediate, and late integration strategies. Finally, it highlights the transformative role of DL in uncovering complex, high‐dimensional patterns for cancer research.

### 3.1 Introduction to Integration Challenges: Data Heterogeneity, Scale, Noise

To enable the examination and understanding of the acquired multidimensional data, all advances in omics approaches necessitate the creation of advanced omics analysis instruments. Large‐scale, high‐throughput research data from multiple observational sources has now been efficiently processed. Related biological information is lost due to the neglect of crosstalk between different molecular entities in the analysis and interpretation of single omics data. Capturing multiple omics data from the same sample provides a unique opportunity to more comprehensively understand the information flow between biological layers. The phrase “data integration” is used extensively in many distinct omics methodologies, such as horizontal data integration, which is the integration of identical omics data from multiple studies or time periods [60, 61]. Vertical data integration is the process of integrating several omics data types that were extracted from the same sample. One of the major areas of current research is vertical data integration, commonly referred to as “multiomics integration,” which is a highly challenging problem.

The complexity and noise of omics datasets, which are inherent in biological data, are among the more specialized ones. Sometimes subtle, relevant patterns involve numerous molecules from various omics layers. Therefore, identifying those patterns across several datasets is a challenging endeavor. Furthermore, for financial reasons, the scarcity of the desired phenotype, a shortage of volunteers, and so on, significant volumes of biomedical data may frequently only be collected on a limited sample of patients when performing multiomics investigations. This causes the number of variables in the dataset to be much larger than the sample size. This problem is known as the curse of dimensionality. Machine learning algorithms tend to overfit these high‐dimensional datasets, which reduces their generalization ability on new data [62]. Since omics can contain a variety of data forms and distributions, their heterogeneity presents another difficulty that needs to be appropriately managed. A metabolomics dataset may have a few thousand variables, but a gene expression dataset often has tens of thousands. This is just one example of how omics datasets may vary greatly in size. These differences in omics might make it difficult to integrate them and lead to an unbalanced learning process.

### 3.2 Early Integration Strategies

Concatenating all of the datasets into a single, sizable matrix is the foundation of the early integration. The number of variables is increased during this procedure, while the number of observations remains constant. This process thus creates a more complicated, noisy, and high dimensional matrix, which makes learning challenging and exacerbates a number of integration issues. Furthermore, the size difference among omics datasets may lead to a learning imbalance since the algorithm ignored the other omics and spent more time learning on the omics with the most variables [63]. Additionally, early integration disregards the unique data distribution of each omics, which may lead machine learning models to identify patterns that are not meaningful and just represent the features shared omics membership. Nevertheless, early integration is still widely utilized because it has several obvious benefits, including as simplicity, ease of implementation, and the ability for machine learning models to immediately expose connections between the various layers by mixing variables from each omics.

The complexity of the composite matrix must be addressed by approaches that employ the early integration strategy, usually by lowering the number of variables using feature selection or dimensionality reduction techniques. Since it is adaptable and strong enough to precisely identify pertinent patterns even in concatenated data, DL has become widely employed in recent years. For example, Xie et al. used clinical and multiomics data to feed the input layer of an artificial neural network, which was then connected to a Cox proportional hazard model to forecast cancer patients’ chances of survival [64]. For a similar purpose, Chaudhary et al. used an autoencoder in place of the popular fully connected neural network to lower the multiomics matrix's dimensionality and extract compact and significant DL‐based variables, which were then clustered using the *k*‐mean approach [65].

One of the most difficult problems with neural networks is their “black box” character, or inability to be interpreted, despite their great degree of adaptability and often better performance on large datasets. Strong predictive models alone are insufficient, especially in biomedical research, where knowledge of the roles played by genes and other molecules in underlying biological processes is essential. Most of these strategies focus on explaining the final decision of the algorithm and identifying biomarkers, but some DL models can directly uncover relevant biological pathways during learning [66]. Furthermore, the early approach enables the inference of heterogeneous networks through the use of techniques like mixed graphical models, which are an extension of Gaussian graphical models, which assume a normal distribution of variables [67]. Depending on the kind of variable, MGM uses either logistic regression or linear regression to regress each variable against every other variable. Another decision tree‐based approach is graphical random forest, which uses all other features as predictors and computes a random forest for each variable [68]. Features are thought to interact with the chosen variable if they are regarded as significant by random forest significance metric.

### 3.3 Intermediate Integration: Multivariate Statistical Models

We define intermediate integration as any technique that may jointly integrate the multiomics datasets without requiring a simple concatenation or previous modification. They often provide newly generated representations that may be used for additional analysis, some of which are omics‐specific and others of which are common to all omics. The multiomics datasets dimensionality and complexity are decreased in this stage. However, they are often employed following feature selection and thorough preprocessing, as dataset variability may render them ineffective. Only a few methods are able to identify partially shared structures—patterns that are shared among some but not all of the omics data.

The premise behind intermediate approaches is that the various datasets have a shared latent space that can disclose the biological mechanisms at play. These techniques have led to the development of joint non‐negative matrix factorization (NMF) and integrative NMF, two expansions of the popular NMF [69, 70]. While integrative NMF employs sample clustering and subtype identification, joint NMF uses the common space to find modules of associated multiomics data. Both approaches infer a common matrix that shows the latent associations between each omics dataset.

### 3.4 Late Integration: Knowledge‐Based and Network‐Based Approaches

The simplest integration technique for managing multiomics datasets is late integration, which involves applying machine learning models independently to each dataset before combining their individual predictions. In contrast to the other methodologies, it does not face the difficulties of attempting to compile many types of data, and its strength comes in its ability to employ easily accessible tools created especially for each omics type. To create a single final prediction for cancer prognosis, Sun et al., for instance, constructed neural networks for every dataset that included gene expression, copy number, and clinical data. They then linearly aggregated these predictions [71]. In order to retrieve the initial classification predictions, Wang et al. employed a more intricate aggregation function. They trained Graph Convolutional Neural Networks on each omics (as well as their corresponding patient similarity networks). Then, using the single‐omics predictions, a cross‐omics tensor was created and sent to a view correlation discovery network, which uses the latent cross‐correlation between omics and the individual omics predictions to get the final class prediction [72]. The inability of such an integration technique to capture interomics interactions and the inability of the many machine learning models to exchange knowledge and use complementarity information between omics at any stage of the learning process are its drawbacks.

### 3.5 Cutting‐Edge Integration: The Role of AI and DL

Artificial intelligence (AI) is playing an increasingly important role in the context of “big data” mining, particularly in precision medicine. Because DL techniques can be used to a wide range of applications and can handle heterogeneous, sparse, noisy, and high‐dimensional single‐cell omics data, they have attracted a lot of interest, especially in the multiomics field [73]. For example, it has been demonstrated that DL techniques are quite successful in tasks like cellular trajectory inference, data imputation, batch effect removal, and dimensionality reduction [74]. Therefore, there has been an increasing emphasis on improving the interpretability of models, especially for uses such as reassembling biological networks and determining molecular control.

Multimodal measures, including proteomics, genomes, and measurements from multiplexed marker‐staining platforms, are likely to be necessary for future pathological diagnosis to provide a whole patient‐specific portrait for tumor precision therapy [75]. The promise of DL‐based AI techniques for digital pathology is encouraging despite the aforementioned difficulties and barriers since AI has a strong feature representation learning capability made possible by algorithm advancements, the collection of large amounts of data, and greater processing power. Following multicenter data validation and improved interpretability, people will have greater faith in AI systems. AI and pathologists working together will enable more precise treatment of tumors.

## 4 A Case in Point: Decoding CRC Through a Multiomics Lens

Here, CRC is presented as an illustrative model to demonstrate the practical application of multiomics approaches. The section first details the genetic and molecular pathology of CRC, then systematically reviews how genomics, transcriptomics, proteomics, metabolomics, and microbiomics each contribute to biomarker discovery and mechanistic understanding, culminating in a discussion on integrated insights and therapeutic guidance.

### 4.1 Pathophysiological Signatures of CRC

The genetic and molecular paradigm that CRC has provided for the evolution of solid tumors has shed light on the concepts of early identification, risk assessment, prevention, and therapy. In this section, we will discuss the pathophysiological processes of CRC in detail, focusing on the genetic changes and cellular biological features that underlie this disease. The underlying pathophysiological signatures of CRC are discussed in detail below.

#### 4.1.1 Genetic Characteristics of CRC

The variability within and between tumors induced by gene alterations during disease start and development has been revealed by thorough genomic study of CRC. The molecular classification of CRC is currently based on microsatellite instability (MSI), KRAS or BRAF mutations, and occasionally chromosome instability (CIN) [76]. Genome stable CRC is those that do not have CIN or MSI [77]. While a sizable fraction of MSI CRC and a minor number of CIN CRC are also CpG island methylator phenotype (CIMP)‐positive, and around 10% of CRC are negative for CIN, MSI, or CIMP, it is most likely the case that CIMP transcriptionally inhibits the DNA repair genes and tumor suppressors in CRC [78].

CIN, which includes 65–70% of sporadic CRC, is characterized by chromosomal structural and numerical abnormalities, such as somatic copy number variations, deletions, or heterozygosity loss [79]. Wnt signaling is present in almost all CIN tumors, and 80% of them have mutational inactivation of adenomatous polyposis coli (APC), a Wnt pathway negative regulator [79]. In 60% of CIN tumors, TP53 is mutationally inactivated or deleted. The loss of p53 function is twofold: it causes CIN directly and creates an environment that is favorable for pathways involving genomic instability. Anaphase bridges observed in early‐stage CRC in humans and spontaneous CRC in animals with telomerase‐deficient p53 mutations indicate that coupled telomere dysfunction and p53 insufficiency is a significant mechanism of CIN [80, 81]. Defects in chromosomal cohesion, centrosomes, microtubule attachment, mitotic spindles, and checkpoints can also lead to aneuploidy and cause mistakes in chromosome partitioning during cell division. A third of sporadic CRC has microdeletions in the MACROD2 gene, which interfere with PARP1's transferase catalytic activity. This leads to aneuploidy, spindle checkpoint relaxation, and a deficit in DNA repair during mitotic entry [82].

Microsatellites, which are DNA sequences with repeated patterns, tend to experience higher rates of mutation compared with other genomic regions. The mutational inactivation of several mismatch repair gene (MMR), such as PMS2, MLH1, MSH2, MSH3, and MSH6, causes the phenotypic expression of deficient MMR, known as MSI. MSH2 controls the identification of mismatches and initiates repair by forming heterodimers with MSH6 or MSH3 [83]. Loss of MMR activity accelerates the development of CRC due to genetic hypermutation. In many Lynch syndrome patients, the immediate upstream gene of MSH2, TACSTD1, which encodes EPCAM, is lost germline, causing in transcriptional read‐through that hypermethylates the MSH2 promoter [84]. The cellular stress, inflammation, and hypoxia in CRC cause MSH3, which corrects mismatched dinucleotides and tetranucleotides, to be mislocalized or suppressed [85, 86]. In conclusion, tumor heterogeneity is influenced by genome instability mechanisms, which can manifest as CIN or MSI, each with unique genetic characteristics. For CRC patients, these designations have significant diagnostic and treatment ramifications.

**Telomerase Reactivation and Telomere Dysfunction**

The shelterin complex and telomeres work together to safeguard and maintain chromosomal integrity [87]. Studies conducted on the telomerase‐deficient mice model further confirmed the significance of telomere dysfunction in CRC [80]. Telomere dysfunction has been associated to the transition from adenoma to cancer in humans, and increased telomere erosion in the intestinal epithelium with aging may indicate a role for a telomere‐based crisis in the early stages of CRC in humans [88].

Apart from CIN, telomere biology is involved in significant mechanisms that govern carcinogenesis, such as inflammation, which is a recognized trigger for CRC development. Inflammatory bowel disease (IBD) patients have an increased chance of acquiring CRC, especially if they have ulcerative colitis (UC) [89]. The cumulative risk of CRC in UC patients who have had the condition for 10, 20, or 30 years is 2, 8, and 18%, respectively [90]. Evidence suggests that CIN following may lead a higher incidence of CRC. This is supported by the observation of accelerated telomere attrition in the colon of UC patients [89]. The intestinal epithelium age‐dependent telomere attrition may play a major role in late‐onset IBD and its recurrence. Telomere disruption can trigger ATM/cABL, which phosphorylates and activates YAP1, causing upregulation of prointerleukin IL‐18 [91]. Furthermore, elevated intracellular reactive oxygen species (ROS) levels hasten telomere attrition and damage, creating a feedback loop of telomere dysfunction–inflammation that may exacerbate genomic instability and eventually lead to cancer [92]. Cells experiencing a crisis may contain extrachromosomal telomere fragments that activate the cyclic GMP–AMP synthase/stimulator of interferon genes (cGAS/STING) pathway, leading to persistent inflammation through a Type I interferon‐mediated mechanism. The relationship between telomeres and cGAS/STING may serve as an additional trigger for inflammation‐related cancers and provide a basis for novel treatment strategies [93].

In terms of mitochondrial biology and oxidative defense, the junction of telomeres and other CRC characteristics is significant. In particular, p53 is activated by telomere disruption and is a regulator involved in mitochondrial biogenesis. In addition to producing free radicals and chemicals such as conjugated dienes, lipid peroxides, and malondialdehyde, ROS may oxidize polyunsaturated fatty acids [94, 95]. Protein oxidation can negatively impact protease fidelity, DNA repair enzymes, and the proteasome system, which removes misfolded and damaged proteins [96]. Many diseases, including CRC, can be caused by this accumulation of defective proteins. There are several reasons why telomerase reactivation is relevant to the pathogenesis of CRC. The confluence of mitochondrial biology and oxidative defense with other CRC characteristics and telomeres is a significant aspect. Protease fidelity, DNA repair enzymes, and the proteasome system, which removes misfolded and damaged proteins, can all be negatively affected by protein oxidation [96]. Many diseases, including CRC, can be caused by this accumulation of defective proteins. There are several reasons why telomerase reactivation is important in the pathogenesis of CRC [97]. While mutations in the telomerase reverse transcriptase gene (TERT) gene promoter are common in many cancers (up to 80% in some malignancies), they are found in only 10% of cases of CRC [98].

The formation of adenomas for carcinomas depends on telomere dysfunction and telomerase reactivation via a variety of pathways. In addition, telomere dysfunction itself has the potential to increase inflammation and reduce ROS defense, which can support carcinogenesis in a number of ways.

#### 4.1.2 Molecular Pathological Characteristics of CRC

Somatic genetic mutations trigger important signaling transduction pathways, which give cancer cells the ability to proliferate. The epidermal growth factor (EGF) receptor (EGFR) and Wnt/β‐catenin pathways are the two main proliferative signaling pathways in CRC.

##### 4.1.2.1 EGFR Signaling Pathway

In CRC, mutations in these signaling components are common. The one exception is EGFR itself, which only mutates in 1% of CRC patients while being overexpressed in over 80% of them [99, 100]. The protein arginine methyltransferase 1 (PRMT1) may methylate R198 and R200 in order to posttranslationally modify EGFR, even when an EGFR inhibitor is present. This increases PRMT1 binding to EGF and the subsequent activation of signaling [101]. In the process of transforming intestinal stem cells into intestinal organoids and promoting the production of APC mice organoids, hepatocyte growth factor (HGF) may entirely replace EGF. Fibroblasts linked to cancer release HGF, a ligand for the mesenchymal–epithelial transition (MET) receptor. These results confirm that MET capacity to avoid EGFR inhibition in CRC [102, 103]. The downstream signaling of EGFR can likewise be activated by human EGFR 2 (HER2) activation. It is noteworthy that activating HER2 mutations allow colon epithelial cells to proliferate without anchoring, and that somatic HER2 mutations and gene amplifications contribute for 70% of instances of CRC [104]. Mutational changes in the downstream signaling components of EGFR determine whether EGFR blocking can continue to support cancer cell survival and proliferation following EGFR inhibition [105]. Approximately 50% of patients with CRC have the rat sarcoma (RAS) activation mutation [106]. It has been demonstrated in recent research that small molecules that covalently attach to the less common G12C mutation can block oncogenic RAS, which was previously thought to be undruggable [107]. In early clinical studies, these inhibitors demonstrated antitumor action; they were most effective against non‐small cell lung cancer and less effective against CRC [108].

About 5% of Stage IV CRC and 10–15% of early‐stage CRC have mutations in the RAS effector v‐raf murine sarcoma viral oncogene homolog B1 (BRAF), which is related to the RAS pathway in CRC. BRAF and RAS mutations are generally mutually exclusive in CRC [109, 110]. BRAF mutation is a poor prognostic factor for CRC [111]. Transcription factors such as NF‐κB and MYC are activated when BRAF triggers mitogen‐activated protein kinase (MEK) [112]. In metastatic CRC, BRAF inhibition by itself was only partially effective because of EGFR‐mediated mitogen‐activated protein kinase signaling. For patients with metastatic BRAF‐mutant cancers, the authorized combination of EGFR inhibitors with BRAF and MEK inhibitors is presently the standard of therapy [113]. In addition, the impact of a drug on host components in the tumor microenvironment should be considered when using certain RAS pathway inhibitors in clinical applications. For example, MEK inhibitors reduce antitumor immunity by inhibiting both cancer cells and T cells, whereas RAS inhibitors are selective for cancer cells and spare T cells [107]. In addition, it is important to note that oncogenic RAS upregulates cytokines that recruit immunosuppressive myeloid cells to interfere with the response to immunotherapy.

Some potential mechanisms that target PI3K/AKT/mTOR pathway activation in CRC include activation mutations in the PI3K component p85, loss of heterozygosity in the PI3K signaling negative regulator PTEN, and amplification of AKT1 [114, 115]. RAS mutations and PIK3CA mutations often coexist, and mutant RAS can also directly interact to trigger PI3K signaling [116, 117]. Increased S6K1 and eIF4E activity caused by mTOR activation supports protein translation and increases cell growth and proliferation [118]. Therapy‐induced suppression of PIK3CA and mTOR, alone and in combination, has demonstrated antitumor efficacy in preclinical models of CRC [119]. Nevertheless, this is not replicated in clinical trials, pointing to a very intricate signaling network in vivo that attenuated the antitumor efficacy of single medicines and necessitating combinatorial targeting of several key pathway components as well as alternate feedback loops.

##### 4.1.2.2 Wnt/β‐Catenin Signaling

Persistent Wnt activation causes CRC, whereas Wnt/β‐catenin signaling preserves normal and malignant cells. The destruction complex governs how much β‐catenin is present in the cytoplasm. β‐Catenin migrates to the nucleus and separates from the destruction complex as a result of the accumulation of Wnt ligands. When TCF or LEF binds to β‐catenin, it activates a number of genes that promote tumor development, including the previously described TERT [120]. The most frequent factors activating Wnt signaling without a ligand change in APC and CTNNB1 are those encoding β‐catenin, which are found in 80 and 5% of CRC patients, respectively. Since Wnt signaling contributes to the maintenance of tumors, it is an excellent target for intervention. APC gene repair leads to cancer cell differentiation, tumor shrinkage without recurrence, and the crypt in the tumor mouse model induced by kras activation‐villus structure returns to normal [121]. APC may be degraded by lysosomal recognition in addition to mutational inactivation caused by β‐catenin‐induced enhanced vesicular trafficking via positive feedback regulation [122]. On the other hand, CRC has the ability to suppress Wnt signaling repressors, which bind β‐catenin to inactive complexes and encourage β‐catenin destruction [123, 124]. The transcriptional transactivation potential of β‐catenin in CRC may be enhanced by RTK‐mediated phosphorylation and upregulation of coactivator proteins linked to the β‐catenin complex [125]. For instance, the interaction between β‐catenin and the histone demethylase JMJD2D, which removes the repressive H3K9me3 mark, thereby facilitating target gene transcription [126].

Ligand‐dependent Wnt signaling is initiated at the Frizzled receptor complex and its coreceptors, LRP5 and LRP6 [127]. Norrin and r‐spondin (RSPO) act as potent Wnt agonists through the frizzled complex [128]. By contributing to the canonical Wnt pathway, RSPO significantly encourages the growth of intestinal crypts. The RSPO receptor leucine‐rich repeat‐containing G protein‐coupled receptor 5 (LGR5) is a marker for intestinal stem cells [127]. While LGR5 stem cells seem to be the favored cell of origin for CRC, more study in this field employing single‐cell omics may be helpful. The E3 ligases have the ability to degrade LGR5, which in CRC leads to high‐grade dysplasia, stimulates the growth of intestinal stem cells, and quickens the course of the disease [129]. In addition, RSPO fusion increases the susceptibility of CRC cells to asparaginase therapy by blocking glycogen synthase kinase‐3β (GSK3β), restricting protein degradation, and lowering the synthesis of free asparagine. Notably, in a minority of patients with stromal‐rich CRC, desmoplastic stromal synthesis of RSPO ligands may make up for the absence of epithelial mutation [130].

**FIGURE 3:**
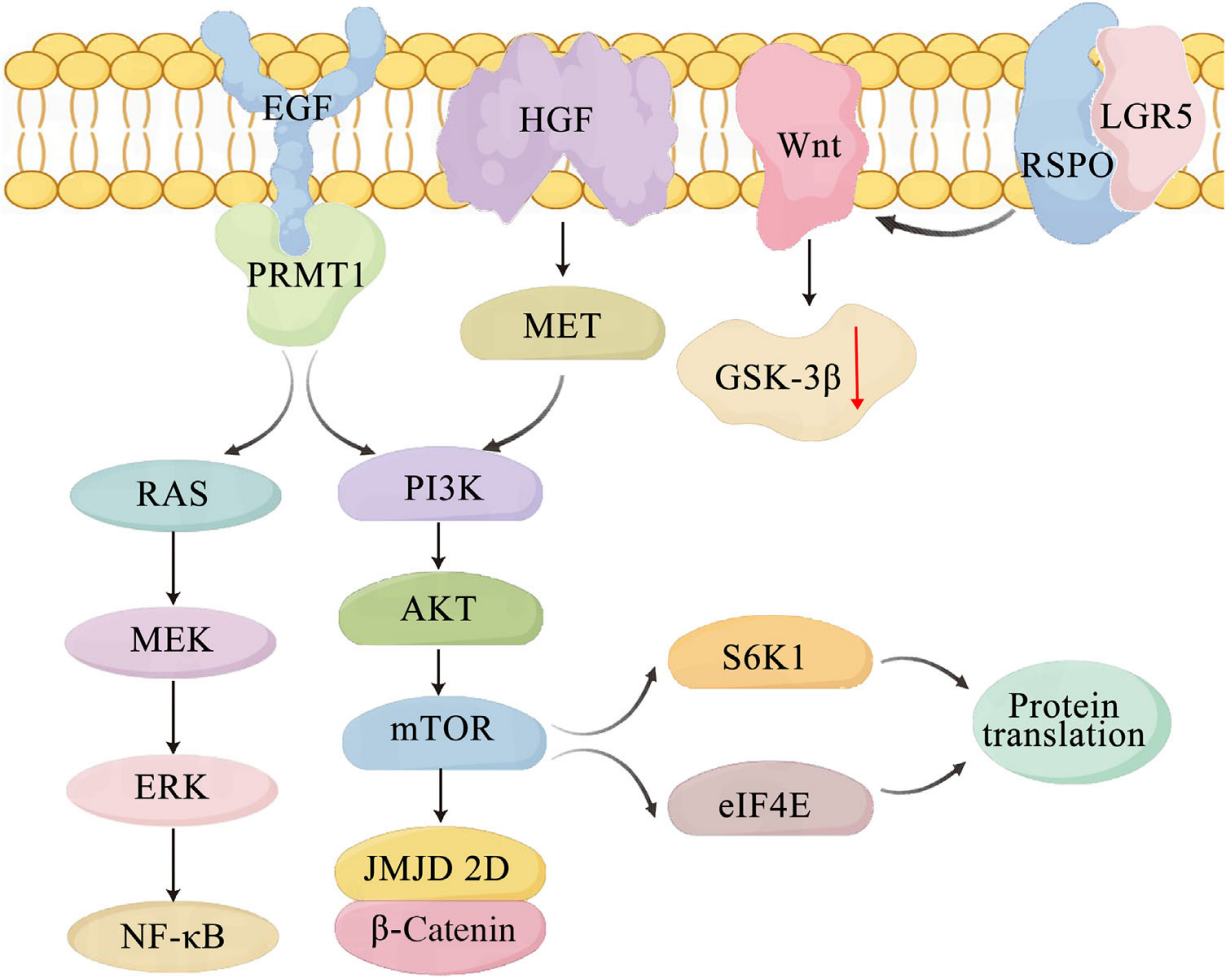
Growth signaling and their interconnection in CRC (by FigDraw). Pathologically dysregulated molecules may be useful therapeutic targets that can meet key requirements for drugs. *Abbreviations*: EGF: epidermal growth factor; HGF, hepatocyte growth factor; RSPO: r‐spondin; LGR5, leucine‐rich repeat‐containing G protein‐coupled receptor 5; PRMT, protein arginine methyltransferase; MET, mesenchymal–epithelial transition; RAS, rat sarcoma; MEK, mitogen‐activated protein kinase; APC, adenomatous polyposis coli.

In summary, almost all CRC has genetic and epigenetic alterations that are maintained by the EGFR and Wnt/β‐catenin pathways. These pathways are also critical in promoting cancer cell growth and other biological characteristics of tumors. New avenues for improving therapy through medication combinations are constantly being opened up by our growing understanding of the processes behind drug resistance (Figure 3).

### 4.2 Unraveling Carcinogenesis: Integrated Insights From Genomics to Microbiomics

#### 4.2.1 CRC Genomics

Numerous significant clinical research problems were addressed by genomic investigations of CRC outcome and recovery. These studies provide fresh approaches to treatment and broaden our understanding of the mechanisms behind CRC. Genetic and epigenetic alterations that occur during carcinogenesis aid in the search for CRC biomarkers [131]. Studies have shown that genetic alterations are a major factor in the development of tumors. As a result, genomics is emerging as a potent tool for identifying genetic markers that enhance our knowledge of cancer and can be used to diagnose and prognosticate the disease. High‐throughput sequencing is a genomic technique used to sequence the DNA of an organism [132]. For instance, Ghatak et al. used differential gene expression analysis in a CRC cohort and validated their results in a clinical cohort. The authors developed a novel biomarker for early diagnosis and prognosis of CRC [133]. When a nucleotide sequence in the genome undergoes a change beyond its original form, it is called an epigenetic change [134]. Epigenetic processes, including DNA methylation, histone modification, and nucleosome placement, govern gene expression. The inhibition in these regulatory processes promotes malignant transformation by impairing gene function [135, 136]. When CRC occurs, the CpG promoter exhibited aberrant methylation. This results in promoter hypermethylation in the genes that encode tumor suppressors and the inhibition of DNA repair genes transcriptional activity. Hypomethylation, or the loss of methylation, is linked to CIN and MSI [137, 138]. The CpG promoter exhibits aberrant methylation during CRC, resulting in promoter hypermethylation in tumor suppressor gene promoters and the suppression of DNA repair gene transcriptional activity. This is accompanied by a loss of methylation, or hypomethylation, which contributes to CIN, MSI, and oncogene activation [139]. In addition, the epigenetic modification of 5mC is associated with CRC. Yang and Qiang et al. both found that genetic variants of the 5mC‐modified gene predicted CRC risk [140, 141, 142]. Currently, 5mC is not considered a viable diagnostic marker for early recognition of CRC, but a growing number of studies have shown that it has a significant impact on the occurrence and development of CRC [143, 144, 145]. Further studies may establish 5mC as a clinical diagnostic biomarker for CRC. Table 1 summarizes the biomarkers discovered in genomic research.

**TABLE 1:** Potential CRC biomarkers were identified in genomics studies of clinical experiments.

Species	Year	Gene	Sample	Findings	References
Human	2022	CBX8, CD96	Tissues	It has been discovered that CBX8 and CD96 play important roles in the development of CRC.	[146]
Human	2021	SOX21	Stool	The relatively high sensitivity and specificity of the SOX21 gene promoter methylation stool‐based test makes it a potential noninvasive biomarker for early detection of CRC.	[147]
Human	2022	MTUS1	Tissue	MTUS1 is a promising biomarker for predicting the diagnosis and prognosis of CRC patients.	[148]
Human	2022	GALR1	Tissue	Epigenetic changes in the GALR1 promoter gradually accumulate during the progression of colorectal tumors and may be a promising biomarker for screening and monitoring CRC.	[149]
Human	2022	SNORD15B, SNORA5C	Tissue	SNORD15B and SNORA5C have tumorigenic effects in colorectal cancer carcinogenesis and are potential diagnostic and prognostic biomarkers for CRC.	[150]
Human	2022	LRRC19	Tissue	In CRC, LRRC19 may be a viable target for prognostic evaluation and early diagnosis.	[151]

*Abbreviations*: CBX8: chromobox 8; CD96: cluster of differentiation 96; SOX21: SRY‐box transcription factor 21; MTUS1: mitochondrial tumor suppressor 1; GALR1: galanin receptor 1; LRRC19: leucine rich repeat containing 19.

#### 4.2.2 CRC Transcriptomics

Researchers have used transcriptomic approaches to assess and examine the amount of RNA expressed in biological systems. It is separated into two categories: noncoded RNA and coded RNA [152]. Transcriptome alterations may occur in CRC due to epigenetic and genomic instability alterations. Coded and uncoded transcripts are analyzed using microarray or RNA sequencing (RNA‐seq). It focuses on factors affecting the transcriptome and aids in the selection of relevant biomarkers from a wide range of RNA species. Many studies have shown that the expression of ncRNA is abnormal in CRC. The study of the stability of ncRNA in stool, plasma, and serum may open new avenues for the development of ncRNA detection techniques, and microRNAs showed to have a major impact on CRC [153]. MiRNAs are tiny RNAs with a length of 21–25 nucleotides that connected to angiogenic, inflammatory, and metabolic processes [154]. Numerous studies have revealed miRNA alterations in CRC [155, 156, 157]. Studies have shown that miRNA‐23a, miRNA‐126, miRNA‐940, and miRNA‐1290 are good prognostic indicators in the initial stages of CRC [158]. Numerous miRNAs were identified as important CRC markers, including miRNA‐192a, miRNA‐29a, miRNA‐19a‐3p, miRNA‐92a‐3p, miRNA‐125b, miRNA‐422a, and miRNA‐223‐3p. Moreover, miRNA‐21 has been extensively studied for the diagnosis of CRC [159]. For example, Allgayer and Dong et al. found that miRNA‐21 is potentially diagnostic for CRC. However, Naidoo et al. found that miRNA‐21 did not play a significant role in the occurrence and progression of CRC. Therefore, the combination of transcriptomics and other omics is important for the study of CRC.

Additionally, unlike RNA‐seq, which measures transcript levels across various cell types, single‐cell RNA‐seq evaluates the transcriptomic status of particular populations of single cells. Thousands of single cells can be handled and barcoded simultaneously in microdroplets and microwells [160]. For instance, prior to linear amplification of mRNA, samples are examined and pooled using the 30 ends of transcripts and the CEL‐Seq barcodes during Quartz‐Seq [161]. In a recent study, transcriptional profiles of 371,223 cells from CRC and nearby normal tissues were extracted from 34 tumors with weak MMR and 28 tumors with strong MMR [162]. One important conclusion of this study is that T cells are arranged in an organized manner inside tumors. The major potential CRC biomarkers identified through transcriptomics research are summarized in Table 2.

**TABLE 2:** Potential CRC biomarkers were identified in transcriptomics studies of preclinical and clinical experiments.

Species	Year	Gene	Sample	Findings	References
Mice	2023	miRNA‐218‐5p	Tissue	MiR‐218‐5p activates the Ras/ERK/c‐Fos signaling pathway to promote CRC development.	[163]
Mice	2024	miRNA‐3655	Tissue	KRAS mutations affect the intratumoral colonization of ETBF in CRC through the miR3655/SURF6/IRF7/IFNβ axis.	[164]
Mice	2025	miRNA‐130a‐3p	Tissue	miRNA‐130a‐3p promotes cholesterol biosynthesis, downregulates AMPK proteins, and activates SREBF2.	[165]
Mice	2025	miRNA‐5692a	Tissue	The miR‐5692a/IL‐8 axis induces an epithelial–mesenchymal transition, which promotes liver metastasis in CRC.	[166]
Mice	2022	miRNA‐4746	Tissue	mRNA‐4746 could serve as a potential prognostic marker and therapeutic target for CRC.	[167]
Mice	2025	miRNA‐423‐3p	Tissue	The miR‐423‐3p/Bim signaling axis promotes epithelial–mesenchymal transition in CRC.	[168]
Human	2018	miRNA‐92a, miRNA‐21	Serum	miRNA‐92a acts as a miRNA and targets Wnt/β‐catenin, PTEN/Akt/FoxO, BMP/Smads related genes, thus participates in CRC progression.	[169]
Human	2014	miRNA‐429	Tissue	miRNA‐429 is a biomarker of prognosis in CRC.	[170]
Human	2017	miRNA‐126‐3p	Tissue	The expression of miR‐126‐3p has been associated with the prognosis of patients with metastatic CRC treated with bevacizumab.	[171]
Human	2018	miRNA‐552	Tissue	High expression of miR‐552 has been associated with a poor prognosis in patients with CRC and may be a potential biomarker and therapeutic target for CRC patients.	[172]
Human	2019	miRNA‐1290, miRNA‐320d	Tissue, plasma	Circulating miR‐1290 and miR‐320d are novel promising biomarkers for early diagnosis of CRC.	[173]
Human	2020	miRNANA‐186‐5p	Plasma	miRNA‐186‐5p has been identified as a potential biomarker to discriminate between CRC patients.	[174]
Human	2020	miRNA‐1539	Serum, tissue	miR‐1539 may be used as a novel potential biomarker for CRC screening and as a predictor of adverse clinical and pathological behavior in tumors.	[175]
Human	2021	miRNA‐449a	Peripheral blood	miRNA‐449a may be useful as a diagnostic and prognostic indicator of CRC.	[176]
Human	2022	miRNANA‐200c‐3p, miRNANA‐1290	Tissue	miRNA‐200c‐3p and miRNA‐1290 measured by PCR can be used as prognostic biomarkers for CRC.	[177]

*Abbreviations*: ETBF: bacteroides fragilis; ERK: extracellular regulated protein kinases; KRAS: Kirsten rat sarcoma viral oncogene homolog; SURF: surfeit 6; IRF7: IFN regulatory factor 7; IFNβ: interferon beta 1‌; AMPK: adenosine 5′‐monophosphate‐activated protein kinase; SREBF2: sterol regulatory element binding transcription factor 2; PTEN: gene of phosphate and tension homology deleted on chromosome ten; BMP: bone morphogenetic protein gene.

#### 4.2.3 CRC Proteomics

Proteins are functional elements that perform and regulate most biochemical activities in living organisms [178]. The proteome is not only a source of potential biomarkers, but also a functional translation of the genome. Advances in technology have made it possible to simultaneously characterize thousands of proteins [179]. Potential biomarkers for stroke were identified using these quickly evolving proteomic methods, which have improved our understanding of disease pathogenesis. Since proteomics is more closely aligned with phenotype than transcriptomics or genome, it has gained popularity as a target for studying CRC biomarkers. For example, Hao et al. used high‐resolution Fourier transform MS to reveal overexpression of dipeptidase 1 in colorectal tumor tissue based on examination of 22 pairs of normal tissues near cancer tissue [180]. Similarly, a separate investigation found that fibroblasts from human and animal tissue were associated with cancer development. The findings of this investigation suggested that the proteins, such as LTBP2, OLFML3, CDH11, CALU, and FSTL1, were potential biomarkers with significant functions in migration and invasion [181]. Blood‐based indicators are among the most promising prospective biomarkers of CRC for early identification and surveillance of CRC due to the relatively simple, noninvasive, and low‐risk acquisition of specimens [182]. Bhardwaj et al. identified a protein panel for the early detection of CRC by profiling five markers in plasma samples from 96 CRC patients and 94 controls using liquid chromatography/multiple reaction monitoring‐MS. The markers were serum paraoxonase lactonase 3, osteopontin, transferrin receptor protein 1, mannan‐binding lectin serine protease 1, and amphiregulin [183].

Proteomics advances show promise for the diagnosis and treatment of CRC [184]. More significantly, a growing body of research showed that hundreds of modification sites and other PTMs were intimately linked to several biochemical processes of CRC [185]. There were several intriguing findings on PTM in CRC, mostly focusing on a few PTMs such as phosphorylation, acetylation, and glycosylation. PTMSs are increasingly being studied in biological processes, but their crosstalk, or coordination of multiple PTMSs, has not been fully appreciated. This is largely because PTMs are highly dynamic, necessitating the use of extremely sophisticated tools to measure co‐occurring PTMs proteome‐wide, as Aggarwal et al. recently reviewed [186]. Several lines of experimental evidence of PTM crosstalk were found in the PTM studies on CRC. One example is PRMTs, which are enzymes that catalyze the methylation of particular arginine sites in target proteins that are downstream. This process was controlled by phosphorylation at particular PRMT sites [187]. Another example is EGFR, a crucial signaling protein involved in a variety of malignancies, including CRC. It has been shown that three PTMs in EGFR—acetylation, phosphorylation, and methylation—interact with one another over the course of CRC carcinogenesis and medication resistance [188]. Many clinical investigations have found hundreds of disease‐associated PTMs, but the field of study on how to translate these findings into clinical practice is still in its infancy [189]. Rarely have extensive clinical trials provided evidence to support the significance of PTMs as biomarkers for a variety of disorders. In addition, proteomics misses genetic and proteome characteristics, as well as individual heterogeneity in patient response to medication, because it studies whole proteins. It is hoped that biomarkers will lead the way toward precision medicine, where the optimum course of treatment is determined by taking individual characteristics into account. The main possible biomarkers of CRC found in proteomics research are summarized in Table 3.

**TABLE 3:** Potential CRC were biomarkers identified in proteomics studies of preclinical and clinical experiments.

Species	Year	Protein	Sample	Findings	References
Mice	2025	N‐acetylmuramic acid	Tissue	N‐acetylmuramic acid, as a new potential biomarker for the prevention and treatment of CRC	[190]
Mice	2023	Fucosyltransferase 2	Tissue	Fucosyltransferase 2 inducesα‐1, 2 fucosylation and inhibits epithelial‐to‐mesenchymal transition and metastasis of colorectal cancer through low‐density lipoprotein receptor‐related protein‐1 fucosylation.	[191]
Mice	2024	MFGE8	Tissue	MFGE8 increases the expression ofαvβ3 on the cell surface to stimulate macrophages and activate the intracellular Src–FAK–STAT3 signaling pathway.	[192]
Mice	2021	USF2, S100A8	Tissue	The USF2/S100A8 axis promotes epithelial–mesenchymal transition and metastasis, while the extracellular S100A8 inhibits the USF2/S1000A8 axis.	[193]
Mice	2025	SIRT2	Tissue	The role of SIRT2 in TME reprogramming and the potential of targeting SIRT2 to make CRC sensitive to immunotherapy.	[194]
Mice	2025	GP73	Tissue	GP73 blockade is a potential therapeutic strategy for reducing liver metastasis in CRC.	[195]
Human	2017	DPEP1	Tissue	DPEP1 may be a driver of the occurrence and progression of CRC and may act as a potential marker of CRC.	[180]
Human	2014	APOE, AGT, DBP	Serum, tissue	APOE, AGT, and DBP are differentially expressed in independent serum and tissue samples from CRC patients.	[196]
Human	2016	MRC1, S100A9	Serum	The upregulation of MRC1 and S100A9 expression in CRC may help determine the mechanism and screening of CRC.	[197]
Human	2017	ACTBL2	Tissue	The higher abundance of the ACTBL2 protein in CRC and its association and differential upregulation in CRC are novel and contribute to the understanding of the occurrence of CRC and may play a role in the development of CRC biomarkers.	[198]
Human	2017	​ MST1	Serum	MST1 is a potential biomarker for early detection, prognosis, and prediction of distant metastases in CRC.	[199]
Human	2017	HSP47	Tissue	HSP47 in CRC may be a new biomarker for predicting metastasis, early recurrence, and poor prognosis.	[200]
Human	2022	CCL2, CCL4	Serum	CCL4 is essential for diagnosing distant metastases in CRC and CCL2 is essential for diagnosing local metastases in CRC.	[201]
Human	2022	COROC1C, RAD23B, ARPC3	Urine	COROC1C, RAD23B, and ARPC3 provide promising urine protein biomarkers for reliable diagnosis and detection of CRC and also suggest potential intervention targets for metastatic CRC.	[202]

*Abbreviations*: MFGE8: milk fat globule‐EGF factor 8; FAK: focal adhesion kinase‌; STAT3: signal transducer and activator of transcription 3; USF2: upstream transcription factor 2; GP73: golgi protein 73; DPEP1: recombinant human dipeptidase 1; APOE: apolipoprotein E; AGT: recombinant angiotensinogen; DBP: DNA binding protein; MRC1: mediator of replication checkpoint 1; S100A9: S100 calcium binding protein A9; ACTBL2: actin beta‐like 2; MST1: mammalian sterile 20‐like kinase 1; HSP47: heat shock protein 47; CCL2: C–C motif ligand 2; COROC1C: coronin, actin binding protein, 1C; RAD23B: RAD23 homolog B; ARPC3: actin‐related protein 2/3 complex subunit 3.

#### 4.2.4 CRC Metabolomics

Metabolomics is an emerging and rapidly growing field that is one of the most reliable tools for studying physiological and pathological states of the body, discovering biomarkers, and analyzing metabolic pathways [203]. Unlike genomics, transcriptomics, and proteomics, it represents the connection between genes and the environment, which enables it to more accurately describe multifactorial diseases [204]. Although biomarkers and metabolites may differ between specimens and CRC levels, they are still useful for diagnosing CRC [205]. ​The use of metabolomics approaches based on blood, urine, stool, and tissue metabolites can identify biomarkers that can be used to distinguish between individuals with early and advanced CRC [206]. Several studies showed a negative association between stool and urine metabolites in patients with advanced CRC. ​The study found 154 metabolites, including those generated by amino acids, polyamine pathways, urea cycle, tricarboxylic acid cycle, and glycolysis. The levels of these metabolites rose as the cancer progressed, with Stage IV showing the most change. ​In addition, Ning et al. revealed 11 upregulated and four downregulated metabolites in urine samples collected from CRC patients and healthy subjects [207]. Another study used GC–MS analysis based on a metabolomics‐based method to investigate the associations between metabolites and health status in healthy persons and CRC patients.

Metabolomics studies of CRC patients found that increased levels of fecal fatty acids, especially oleic acid, can be used to screen for CRC [208]. Recent studies using proton NMR on CRC tissue and stool showed that butyrate is downregulated in CRC tissue and stool. Meanwhile, alanine, lactate, glutamic acid, and succinic acid are also upregulated [209]. A study using UPLC–MS analysis of stool samples from CRC patients showed significant changes in the levels of sphingolipids and cholesterol esters [210]. Due to the significant contribution of metabolomics to drug discovery, metabolite biomarkers from natural compounds based on UPLC MS have also played an important role in disease treatment. Pharmacodynamic metabolomics studies using mouse serum showed that flavonoids and anthraquinones play a role in the treatment of CRC [211]. To date, no single omics approach provided sufficient information to demonstrate detailed molecular mechanisms and validate biomarker signatures. Table 4 summarizes the main CRC biomarkers identified in metabolomics studies.

**TABLE 4:** Potential CRC were biomarkers identified in metabolomics studies of preclinical and clinical experiments.

Species	Year	Metabolite	Sample	Findings	References
Mice	2024	Oleic acid, allocholic acid	Plasma, fecal	A system consisting of 17 plasma metabolites was established and CRC was accurately diagnosed in validation.	[212]
Mice	2023	Butyrate	Tissues	Butyrate can prevent the development of colorectal tumors and enhance the efficacy of anti‐PD‐1 by inducing functional CD8+T cells.	[213]
Mice	2022	Lysophosphatidic acid	Tissues	Elevated lysophosphatidic acid has been linked to the development of colorectal tumors.	[214]
Mice	2025	Bile acid, ursodeoxycholic acid	Tissues	Bile acid metabolism and promotion of ursodeoxycholic acid production are associated with CRC.	[215]
Mice	2024	Linoleoyl ethanolamide	Tissues	​Abnormal levels of linoleic ethanolamide were associated with CRC.	[216]
Mice	2024	Short‐chain fatty acids, sphingolipid, and glycerophospholipid	Tissues	The metabolic levels of short‐chain fatty acids, sphingolipids, and glycerophospholipids are associated with CRC.	[217]
Human	2018	Fatty acid	Fecal	The metabolic profile of fecal fatty acids changes in colorectal cancer patients, suggesting that fecal fatty acids may serve as a biomarker for CRC screening.	[208]
Human	2019	Acetate	Fecal	Acetate has the highest diagnostic performance for CRC, with an AUC of 0.843 in the training set and good prediction in the validation set.	[209]
Human	2012	Aspartic acid	Serum	Based on GC/MS, serum metabolomics has value for early detection of CRC and may become a new screening method for CRC.	[218]
Human	2014	Glycochenodeoxycholate	Serum	There is a positive association between the serum bile acid metabolite chenodeoxycholic acid and CRC in women.	[219]
Human	2017	Tryptophan, palmitoleic acid, lysine, 3‐hydroxyisovaleric acid	Blood	Tryptophan, palmitoleic acid, lysine, and 3‐hydroxyisovaleric acid were identified as potential biomarkers of CRC.	[220]
Human	2019	Taurine, alanine, 3‐aminoisobutyrate	Urine	For CRC patients, taurine, alanine, and 3‐aminoisobutyrate were effective discriminators in several receiver operating characteristic curve assessments.	[221]
Human	2022	Glutamine a	Serum	CRC characteristics are associated with glutamine addiction.	[222]
Human	2022	N‐nitrosamines	Plasma	It has been shown that the effect of N‐nitrosamine on blood exposure levels in colorectal cancer patients may serve as a potential new early diagnostic biomarker and therapeutic target for CRC.	[223]
Human	2023	Tryptophan, bile acids, choline	Fecal	​​CRC is associated with increased metabolism of tryptophan, bile acids, and choline.	[224]

#### 4.2.5 CRC Microbiomics

Microbiomics is an emerging field of omics techniques that study symbiotic or pathological relationships between microbial communities [225]. The gut microbiome of the human body consists of microorganisms and their genetic material [226]. Different approaches were used by different groups to investigate gut microbiome markers in CRC. Shot‐gun metagenomic sequencing and amplification and sequencing of the V1, V2, and V4 regions are two techniques used to enrich 16S rRNA for variable regions in stool DNA [210]. Several qPCR techniques were employed to measure the abundance of target microbial genes in interesting samples [227]. The treatment of CRC found to be significantly influenced by gut microbiomes. The gut microbiome may be useful for biomarker‐based screening, diagnosis, and/or prediction [228]. A recent study that tested blood and tissue samples from 33 cancer patients showed that the blood contained DNA from specific gut pathogenic bacteria that could be used to distinguish between types of tumors [229]. ​In addition, studies of the gut microbiome and its metabolites were linked to CRC. The correlation analysis between gut microbiome and metabolomics showed promising potential in the prevention, treatment, and diagnosis of CRC [230]. For example, Chen et al. investigated the metagenomic and metabolomic composition of serum collected from normal patients, colorectal adenomas and CRC patients. The results found a total of 885 different metabolites in the serum related to gut bacteria. Eight replicable serum metabolites were identified and used to develop classification diagnostic models for healthy/colorectal adenoma and healthy/CRC [231].

When microbial metabolites from CRC patients were analyzed using the GC–MS technique, the results revealed that acetate was present in high proportions whereas butyrate and ursodeoxycholic acid were found in low concentrations. Another GC–MS metabolomic study identified 19 differentiating metabolites in CRC tissue. Additionally, pathway enrichment analyses showed that patients with CRC show a significant disruption of multiple metabolism pathways, including the metabolism of carbohydrates, short‐chain fatty acids (SCFAs), and secondary bile acids [232]. NMR‐based studies of CRC tumor tissue and stool showed a decrease in butyrate levels in CRC patients. Stool and tissue samples from healthy people had an AUC of 0.692 and 0.717, respectively, for the diagnosis of CRC. Fecal acetate has the highest AUC (0.843) of any indication when it comes to diagnosis [209]. Microbiome and metabolomics studies in CRC suggested that concentrations of amino acid metabolites associated with microorganisms may be a significant factor in CRC diagnosis [233]. As these examples demonstrate, future clinical screening for CRC could benefit from some metabolites of the microbiome. In the treatment of CRC, gut microbiomes have demonstrated a major significance. For instance, the gut microbiome may be useful for screening, diagnosis, prognosis, and/or predictive biomarkers. Alternatively, it could be a modifiable factor influencing the prevention or effectiveness of systemic CRC treatment [225]. A screening marker for asymptomatic people with high‐risk adenomas or CRC is the gut microbiome. For instance, the Fusobacterium nucleatum bacteria can be used as a screening biomarker in fecal samples from patients with CRC and adenomas. Based on metabolic markers and genotoxic metabolites of particular strains, early detection and screening for CRC may also be feasible [234]. The main CRC biomarkers found in microbiomics studies are summarized in Table 5.

**TABLE 5:** Potential CRC biomarkers identified in microbiomics studies of preclinical and clinical experiments.

Species	Year	Biomarkers	Sample	Findings	References	
Mice	2023	Roseburia intestinalis	Plasma, fecal	Roseburia intestinalis is a potential adjuvant to augment the efficacy of anti‐PD‐1 against CRC.	[213]
Mice	2022	Marseille‐P5997, Alistipessp.5CPEGH6, parabacteroides distasonis	Fecal	Marseile‐p5997, Alistipe SP.5CPEgh6, and Parabacteroides distasonis exhibited impaired intestinal barrier function.	[214]
Mice	2025	Akkermansia muciniphila	Tissues	Akkermansia muciniphila has been shown to have the effect of alleviating the intestinal microbiome imbalance caused by CRC.	[215]
Mice	2024	Muribaculaceae, Akkermansia, and Ileibacterium, reduced Fusobacterium	Tissues	Muribaculaceae, Akkermansia, Ileibacterium, and reduced Fusobacterium may be involved in inhibiting the initiation and development of CRC.	[216]
Mice	2023	Pseudobutyrivibrio xylanivorans, Eubacterium limosum, Aeromonas veronii, Campylobacter jejuni, Collinsella aerofaciens, Peptoniphilus harei	Tissues	Pseudobutyrivibrio xylanivorans, Eubacterium limosum, Aeromonas veronii, Campylobacter jejuni, Collinsella aerofaciens, and Peptoniphilus abundances have been linked to CRC.	[235]
Human	2017	Fusobacterium nucleatum, Bacteroides clarus, Roseburia intestinalis, Clostridium hathewayi	Stool	Fusobacterium nucleatum, Bacteroides clarus, Roseburia intestinalis, Clostridium hathewayi combination sensitivity 77.7%, specificity 81.5%	[236]
Human	2020	Actinomycetes	Stool	Actinomycetes in the gut may have positive clinical implications for CRC patients.	[237]
Human	2021	Fusobacterium nucleatum	Stool, serum	Fusobacterium nucleatum may be used as a potential biomarker for early diagnosis of CRC.	[238]
Human	2021	Desulfovibrio desulfuricans	Saliva	A clinical nomogram‐based model that takes into account factors including age, sex, oral hygiene index, and salivary desulfovibrio desulfuricans level can be used to predict the risk of CRC.	[239]
Human	2021	Parvimonas micra, Peptostreptococcus stomatis, Fusobacterium nucleatum, Akkermansia muciniphila	Tissue	Parvimonas micra, Peptostreptococcus stomatis, Fusobacterium nucleatum, and Akkermansia muciniphila as a four‑bacteria biomarker panel of CRC.	[240]
Human	2022	Bacteroides clarus	Stool	This reveals the widespread presence of CRC related microbiota in the population, which may serve as a target for CRC diagnosis and treatment.	[241]
Human	2022	Aspergillus rambellii	Stool	Multicohort metagenomic analysis has linked CRC to altered microbiome signatures and enriched pathogenic Aspergillus rambellii.	[242]

### 4.3 Microbial Metabolites as Key Regulators and Biomarkers in CRC

CRC is the third leading cause of mortality globally, yet the exact molecular mechanism behind it is still unclear. While there is a strong hereditary component to CRC risk, internal and external exposures, as well as their interactions with genetic variables, also have an impact on the pathophysiology of CRC. The advent of omics approach, which is utilized for the objective screening of biomarkers such as genes, transcripts, proteins, metabolites, and microbiomes. In addition, omics approach has recently proven beneficial to the field of CRC research [243, 244, 245]. It may be possible to identify novel biomarkers for CRC screening and diagnosis through an application of omics approach. Less or noninvasive methods for diagnosing CRC are particularly offered by high throughput multiomics approaches, such as transcriptomics, proteomics, microbiomics, and metabolomics. Each method has a unique benefit for finding new CRC biomarkers for diagnosis. For example, genomics is a highly effective way to assess the hereditary risk of the disease and the vulnerability of CRC. However, it has limited diagnostic potential because DNA sequences rarely correspond exactly to phenotypes due to epigenetic, posttranscriptional, and posttranslational changes. Given their closer ties to the physiological states of organisms, transcriptomics and proteomics have enormous therapeutic promise. However, their diagnostic power is inferior to that of metabolomics, which can rapidly and precisely define the phenotypic and metabolic pathways of an organism. Additionally, metabolomics may assess the interaction between gut bacterial metabolites and the host, which is a crucial stage in the development of CRC [246]. Numerous studies have demonstrated the critical role that gut microbial populations and microbial metabolites play in the development of CRC [247]. ​In addition, the distribution of gut microbiota and metabolites can be rapidly updated with dietary changes, making them more suitable for therapeutic interventions for CRC progression. Elucidation of the role of microbial metabolites will provide a new paradigm for CRC diagnosis, prevention, and treatment. The main CRC potential biomarkers found in metabolomics and microbiomics studies are summarized in Table 6.

**TABLE 6:** Potential CRC biomarkers were identified in metabolomics and microbiomics in clinical studies.

Species	Year	Biomarkers	Sample	Application	References
Human	2013	Acetate, butyrate, and ursodeoxycholic acid	Stool	Revealing some potential relationships between fecal metabolites and CRC	[248]
Human	2018	S‐adenosyl‐l‐homocysteine	Colon tissues	Helps distinguish between normal CRC and tumor sites and supports our understanding of the mechanisms of carcinogenesis	[249]
Human	2019	Branched‐chain amino acids, phenylalanine, and bile acids	Stool	Accurate prediction of early development of CRC	[255]
Human	2023	Transition from acetic acid/acetaldehyde metabolism to acetyl CoA production	Stool	Distinguishing early‐onset CRC from late‐onset CRC	[224]
Human	2024	Butyric acid	Stool, serum	To distinguish between colorectal adenocarcinoma and CRC types	[256]

Some metabolites derived from microbial communities were associated with CRC, and the discovery of diagnostic biomarkers for CRC from microbial metabolomics is an active area of research. In the CRC cohort, several studies have identified microbial metabolites as screening biomarkers. For example, a microbiome and metabolome profiling study revealed that CRC patients had lower levels of butyrate and UDCA and greater levels of acetate [248]. Another study revealed that CRC and normal tissue samples differed in function and taxonomy, and that S‐adenosyl‐l‐homocysteine was more abundant in tumor tissue. These findings raised the possibility that microbiome dysbiosis might lead to metabolic variability at various locations [249]. Fecal samples from CRC patients had greater concentrations of amino acids, whereas healthy people had lower levels of polyunsaturated fatty acids, monounsaturated fatty acids, and ursodeoxycholic acid. The correlation analysis revealed that Escherichia coli and obese rumen bacteria were linked to an increase in free fatty acids and glycerol in feces. These correlations may aid our understanding of the role played by microorganisms in the tumor microenvironment and motivate future more mechanistic studies [244]. However, in a study of fecal metabolomics, the concentration of SCFAs did not show significant changes with colon adenoma, cancer, normal, and cancer treatment, suggesting that SCFAs in fecal do not predict CRC [250]. The reason for these divergent results may be attributed to a number of factors, such as sample type, metabolomics analysis platform and strategies used, and the limited number of patients in each cohort.

CRC is known to be a complex physiological process that makes its reversal extremely difficult. Therefore, the treatment of CRC must employ a comprehensive multiomics approach to discover the relevant pathological processes of the cancer and control them in a variety of treatments. The KRAS gene mutation stands as one of the most successful biomarkers in precision medicine for CRC. Its discovery stems from an in‐depth understanding of the EGFR signaling pathway. Early clinical trials revealed that only patients with wild‐type KRAS benefited from treatment with anti‐EGFR monoclonal antibodies (such as cetuximab and panitumumab), while those harboring KRAS mutations experienced no benefit and even adverse effects [251]. Moreover, Immunoscore exemplifies the successful translation of multiomics concepts into a clinical application tool. Its discovery stems from identifying the prognostic correlation between the density of specific immune cells (CD3+ and CD8+ T cells) in tumor cores and invasive margins [252]. This finding was subsequently robustly validated through large‐scale, multicenter international studies involving thousands of patients. The key to its success lies in achieving standardized and automated technology that translates complex tumor immune microenvironment characteristics into a stable, reproducible quantitative score [253, 254]. This score provides independent prognostic information beyond traditional TNM staging systems, aids in identifying patients with low risk of recurrence after Stage II CRC surgery, offers critical guidance for adjuvant chemotherapy decisions, and is currently being explored for its predictive value. In addition, the journey from multiomics biomarker discovery to clinical utility is exemplified by several ongoing clinical trials. These trials aim to validate the diagnostic, prognostic, and therapeutic relevance of omics‐derived signatures. For instance, the success of KRAS mutation status in predicting response to anti‐EGFR therapy is now standard of care, originating from genomic and transcriptomic insights [251]. Building on this, current trials are exploring more complex multiomics signatures. Immunoscore, which quantifies CD3+ and CD8+ T cell infiltration in the tumor microenvironment based on immunohistochemistry (a form of spatial proteomics), has been validated in large international cohorts (NCT03026140, NCT04158458) and is moving toward routine clinical use for staging and adjuvant chemotherapy decisions in Stage II/III colon cancer [252, 253, 254].

### 4.4 Multiomics in Guiding CRC Therapy and Monitoring Response

One appealing tactic for lowering the cancer burden is cancer prevention. Dietary modification is an effective way to lower the risk of CRC, according to epidemiological studies [257]. A high‐fiber diet was found to promote the generation of SCFAs by microbes in a group of patients with advanced colorectal adenoma and was linked to a lower risk of cancer [258]. On the other hand, changes in microbial metabolite composition induced by a high‐fat diet have also been demonstrated in another cohort of dietary exchange experiments [259].

In terms of preventing CRC, direct supplementation of microbial metabolites has also demonstrated promise. Given the encouraging outcomes of preclinical studies, butyrate supplementation appears to be a viable preventive approach for CRC. It has been demonstrated that butyrate increases the effectiveness of radiation in preclinical patient‐derived CRC organoid models. This finding raises the possibility of butyrate being used in clinical settings in conjunction with other cancer treatments [260]. Regarding polyamines, prospective studies on dietary supplements showed that a 39% increased risk of colorectal adenoma was linked to intake of polyamines over the median level in the general population [261]. Nevertheless, dietary polyamines did not appear to be linked to an elevated risk of CRC or death specifically related to CRC in another cohort of postmenopausal women at average risk [262]. The relationship between dietary polyamines and CRC risk has to be further investigated. A comprehensive study of the safety and efficacy of microbial metabolites is still lacking, but it provides a new paradigm for the prevention and treatment of CRC. In the future, supplementing with advantageous metabolites may show to be a practical approach to enhance CRC treatment and surgery.

In conclusion, a great deal of research in the field of cancer research using multiomics technologies has made it more evident that these technologies have the potential to provide significant insights into the intricate molecular changes that characterize the cancer. ​In addition, multiomics applications can be extended to other diseases, providing important information for better understanding the mechanisms behind these diseases and creating possible treatment approaches.

## 5 Toward Clinical Translation: Multiomics in Oncology Precision Medicine

This section explores the pathway from multiomics discovery to clinical application. It discusses the development of diagnostic, prognostic, and predictive biomarker panels, evaluates the role of DL in cancer classification and outcome prediction, and addresses the challenges and opportunities in translating multiomics insights into personalized treatment strategies.

### 5.1 Discovery of Novel Diagnostic, Prognostic, and Predictive Biomarker Panels

The goal of precision medicine is to use advanced omics testing to customize specific medical services for patients. A customized approach to patient care that goes beyond the conventional one‐size‐fits‐all model of medicine is known as precision medicine. The role of multiomics approaches in predicting complex diseases in medicine has recently been highlighted [263]. For example, a recent study used a DL technique to find pharmacological combinations that work well together to treat COVID‐19. The research showed that a DL model can predict medication–target interactions and drug–drug combinations based on molecular structures. It can also identify the most synergistic drug combination for the fast‐spreading SARS‐CoV‐2. [264]. While single‐omics level studies are crucial in understanding disease processes, most single‐omics level data consist of transcriptomics and genomics, and there is insufficient data to attempt to integrate diverse omics data. Genes identified from genomics, transcriptomics, and proteomics studies can be used to construct disease‐related networks at each omics level, and network‐level overlaps can be calculated. Integration of multiomics data, such as microbiome, metabolome, proteome, transcriptome, and genome, is largely lacking.

Multiomics analysis is increasingly being used in biomedical research to assist in understanding the intricate interactions between the molecular levels. However, integrating these different datasets still poses challenges. DL methods are now widely used for disease risk assessment [265]. DL approaches are helpful in generating hypotheses to understand patient stratification and disease course, as well as in discovering biological processes and how they affect disease risk in multiomics data [266]. One tool that employs DL to integrate a large number of biological features is called DeepProg. It may be used to predict cancer prognosis and survival rates [267]. The use of DL approaches to combine multiomics data with deep cohort phenotypes provides a novel approach for deeper functional insights and accurate diagnosis of complex diseases. Ma et al. is the first attempt to reveal the common states of faecal microbiome dysbiosis and metabolome dysregulation in late‐onset and early‐onset cancer patients by leveraging multiomics data. It was found that Fusobacterium nucleatum enrichment and SCFA depletion, including reduced microbial GABA biosynthesis and a shift in acetate/acetaldehyde metabolism toward acetyl‐CoA production characterizes LO‐CRC [224]. A large‐scale clinical study conducted a genome‐wide association study meta‐analysis of 100,204 cancer cases and 154,587 controls of European and East Asian ancestry, identifying 205 independent risk associations, of which 50 were unreported. Cross‐tissue analyses indicated that over a third of effector genes most likely act outside the colonic mucosa [268]. Moreover, multiomics analysis can be utilized to study host–microbiome correlations in eoCRC. The distinct host–microbiome correlations for urea cycle in eoCRC may offer opportunities for therapeutic interventions [269].

Personalized risk assessment and therapy development may now take use of new potential to uncover the genetic foundations of target endotypes, thanks to the availability of omics data and integrated omics approaches. Furthermore, statistical approaches to include multiomics data are developing to offer critical understandings of the pathophysiology of allergic disorders [270]. As research develops, identifying and creating treatment plans for certain allergy subtypes will depend heavily on knowing a person's distinct racial lifestyle, environmental exposure, and data‐driven DL frameworks and integrations based on multiomics. This approach will help in the development of precise treatment plans for different subtypes cancer [271].

The age of single‐cell and spatial omics has been brought about by technological advancements that make it possible to gather and combine data from individual cells. Finding cancer biomarkers and potential treatment targets is essential in biological research on cancer. Innovative approaches to cancer diagnosis and treatment, as well as a deeper understanding of the disease process, can be developed using single‐cell and spatial multiomics data. Above all, future medical practice might be drastically altered by the well‐established techniques of single‐cell and spatial multiomics.

### 5.2 DL‐Based Multiomics Analysis in Cancer Diagnosis, Classification, Target Identification, and Grading

In the age of precision oncology, a variety of omics technologies have been widely employed to diagnose and categorize cancer subtypes, including transcriptomics, proteomics, metabolomics, and genomes. Large amounts of data from many sources may be processed and integrated by AI algorithms, which aids physicians in the diagnosis and classification of cancer kinds. Deep neural networks (DNNs) are strong algorithms that may be used to categorize cancer kinds, diagnose tumors, and determine cancer grades [272]. When it comes to diagnosing malignant tumors, DNN‐based models have demonstrated exceptional accuracy. For example, they can use digital histopathology slides to differentiate between cancerous and normal cells [273, 274]. ​This method accurately diagnoses the type and grade of cancer and distinguishes tumors from normal tissue. Detecting metastases in breast cancer lymph nodes using whole‐slide pictures with hematoxylin–eosin staining is one of the most effective applications of this method in tumor detection, showing more efficiency and accuracy than pathologists' diagnoses [275]. In this competition, the AI system's area under the receiver operating curve (AUC) was 99.4%, a substantial increase above the 11 pathologists 81.0% AUC. Driver gene mutations, whole‐genome duplication, copy number changes, and gene expression levels were shown to be strongly connected with histopathological characteristics in a recent AI‐based multiomics pan‐cancer study [276]. These researches demonstrate the ability of DL‐based pathology analysis to accurately identify different cancer types and even anticipate genetic changes associated with cancer.

High‐throughput technology advancements have led to a shift in cancer categorization from the conventional “morphological” approach to the new “molecular classification” era [277]. Diagnoses and classifications of cancer subtypes have been made using molecular characteristics taken from multiomics technology. However, multiomics technologies frequently provide data with multidimensional information and vast quantities. Large datasets may be processed effectively by DNN‐based algorithms, which can also combine several datasets for the detection and classification of cancer. For example, using DNN techniques, Sun et al. created a genomic‐DL model that used WES data from 6083 tumor samples from 12 TCGA cancer types and 1991 healthy tissues to identify genomic point mutations [278]. In a promising study, Capper et al. created a machine learning system to categorize tumor kinds using DNA methylation patterns and contrasted the precision of the diagnoses with pathologists’ histological diagnoses. The diagnostic accuracy of the AI algorithm was assessed in this study using 1104 test tumor cases that pathologists had identified using histological or genetic methods. Although a portion of the instances (15.5%) were classified as tumors, the machine learning‐based classifier agreed with the pathologist's assessment for 838 of these test cases. The machine learning classifier's diagnosis did not match the pathologist's diagnosis in 139 of the test cases [279]. However, further molecular studies revealed that the classifier actually correctly predicted 93% of those mismatched instances. A related study also confirmed the efficacy of machine learning‐based classification based on copy number and DNA methylation for cancer detection [280].

### 5.3 DL‐Based Multiomics Analysis in Predicting Cancer Prognosis and Treatment Response

In recent years, AI algorithms have incorporated multiomics data, including transcriptomics and genomes, into risk prediction models to guide therapy and forecast the likelihood of tumor relapse and treatment response [281]. Classifiers such as MesoNet, decryption scoring, and molecular prognostic scoring (mPS) systems can provide doctors with more accurate and appropriate treatment strategies [279, 282, 283]. For example, Erho et al. showed that a Decipher score genomic classifier trained just on 22 genomic expression profiles could accurately predict prostate cancer patients’ postprostate metastases [282]. Similarly, Shimizu et al. developed a universal molecular prognostic model based on AI techniques, known as the mPS system, that accurately predicts the survival of patients with breast cancer by evaluating 23 prognosis‐related genes [283]. The AI algorithm is able to extract prognostic data from the whole slide for survival and outcome prediction, including lymphocyte counts, lymphocyte spatial distribution, chromatin patterns, and cell subtype proportions. Using digitized whole‐slide pictures, Courtiol et al. created MesoNet, a deep convolutional neural network‐based model that can predict mesothelioma patients’ overall survival on its own (without the help of pathologists) [279]. Other studies have confirmed that AI algorithms can provide accurate risk ratings for a variety of cancers, such as CRC, brain tumors and breast cancer, etc., when paired with information from whole‐slide photos [284, 285, 286].

Compared with prognosis, AI algorithms forecast pharmacological treatment response with greater clinical relevance, assisting physicians in prescribing more effective treatments. Immunotherapy and targeted therapy have emerged as the most popular cancer treatment modalities over the last 20 years, with promising clinical results [287]. However, different individuals react differently to particular medications because of complicated tumor heterogeneity and genetic changes. The response to immunotherapy and targeted therapy was well predicted by AI‐based multiomics analysis, as demonstrated by a number of recent research. A predictor for forecasting the response to immunotherapy is the MSI test, which is based on genomics. Histopathological phenotypes that exhibit high levels of lymphocyte infiltration and mucinous differentiation, such as MSI tumors, are associated with genetic changes in cancer. For the purpose of predicting MSI for cancer patients who have not had genetic testing done, several studies have combined AI with histological imaging data from tumor tissues of malignancies, including CRC, stomach, and endometrial cancer [288, 289, 290]. To forecast bladder cancer clinical outcomes, Xu et al. created an AI‐derived genetic characteristic (AIGS). The findings demonstrated that patients who were responsive to immunotherapy or other treatment approaches might be reliably identified using AIGS [291]. These studies showed that AI algorithms that use multiomics data have potential for assessing how well cancer patients respond to treatment.

## 6 Current Challenges and Limitations

As discussed in the previous sections, AI techniques are widely employed at various touchpoints throughout the clinical cancer care route and have effectively addressed the complexity and heterogeneity of multiomics data. Even though AI‐based multiomics analysis has advanced significantly, there are still numerous hurdles to overcome before it can be used in precision medicine.

### 6.1 Data Scarcity

Large datasets are typically required for AI techniques to ensure sufficient robustness. However, it can be challenging to obtain enough high‐quality data in precision oncology, which could lead to unconscious biases. High‐quality samples may be obtained by the use of precise sampling techniques, such as image‐guided tissue extraction and three‐dimensional molds created from tumor morphology [292, 293]. Although there are still obstacles to overcome before these techniques are expanded, interinstitutional data sharing and high‐throughput technology may be the simplest means of achieving large‐scale and addressing the shortcomings of data scarcity. Sharing data between institutions can greatly minimize the need for new trials, but it can also increase biases because of data heterogeneity, which might restrict how easily the results can be interpreted. Additionally, one of the main causes of AI systems failure in clinical trials is variability in cross‐institutional data [294]. Importantly, maintaining patient privacy is the fundamental tenet of interinstitutional collaboration [295]. Additionally, current research on transfer learning seeks to enhance the performance of particular students by transferring their prior, broadly applicable information from other distinct but related students. Transfer learning has been effectively used to improve cancer diagnosis and classification as well as therapy response prediction, and it provides a great way to get around the lack of data [296, 297].

### 6.2 High Heterogeneity and Complexity of Multimodal Data

AI‐based multiomics analysis has mostly been used to combine data from transcriptomics and genomes, despite several significant advancements. To address the complex biological issues that underlie oncology disorders, further layers of information must be added. These layers include multilayer regulatory programs that are mediated by different epigenomic alterations, transcription factors, and noncoding RNAs. Furthermore, new spatial omics techniques maintain tissue architecture, which gives researchers even more leverage as they work to gain a thorough grasp of cellular communities and intercellular communication [298]. To speed up cancer research, efforts are being made to develop extensive multimodal data resources that investigators may use. One such initiative is Cancer Moonshot Research Initiatives, which aims to gather a variety of omics and nonomics data in order to build a complete database. How to model multimodal interactions explicitly is the next technological problem. Finding a way to spatially match ex vivo data with in vivo imaging is another critical difficulty. This is particularly relevant when examining the biological association of multimodal characteristics. In order to handle the complexity and variety included in multimodal data, these issues encourage the development of innovative AI approaches.

### 6.3 Lacking Interpretability and Repeatability

The absence of interpretability and repeatability in AI‐based multiomics analysis, which is crucial for fostering therapeutic usefulness, is another difficult problem. We are currently unable to fully comprehend how AI‐based models generate such predictions due to the complexity of cancer, intrinsic differences between modalities, and AI's black‐box nature [299]. This problem prevents AI‐based multiomics techniques from being clinically translated from the lab to the bedside. Thankfully, there have been more attempts to improve interpretability, and a number of methods—including saliency maps, hidden‐state analysis, variable significance measures, and feature visualizations—have been put out to clarify the reasoning behind AI predictions [300]. These methods will assist in gaining mechanistic insights into intricate AI‐based models and provide insightful justifications for patient‐specific forecasts. A number of solutions, such as using a more complicated algorithm or averaging results from several models, attempt to address the issue of repeatability, but they do not appear to address it at its core. To a certain degree, however, adhering to the standard principles can ensure the repeatability, transparency, and scientific rigor of the AI model [301].

In conclusion, multiomics technologies have a lot of potential for precision oncology as they offer a full picture of cancer behavior through detailed profiling. AI has the ability to leverage data from several sources, which can expand our knowledge of cancer biology and lead to more precise cancer diagnosis and treatment approaches. Significant efforts are being made to solve these problems and advance the clinical translation of AI‐based multiomics analysis, despite the fact that there are still numerous obstacles to overcome. As more and more multiomics data are produced in clinical settings, we anticipate that AI will play an increasingly crucial role.

## 7 Future Perspectives: The Next Frontier in Cancer Multiomics

This section outlines emerging directions in multiomics technologies and their anticipated impact on oncology. It covers the potential of single‐cell and spatial multiomics to deconstruct tumor heterogeneity, the utility of organoid models and longitudinal profiling, and the importance of collaborative ecosystems and open science in driving the next wave of discoveries.

### 7.1 Single‐Cell Multiomics: Deconstructing Intratumoral Heterogeneity

Tissues and organisms are highly ordered structures composed of multiple cell types. Conventional experimental techniques, such as histological staining, distinguish between various cell types according to their shape, location, and functions within tissues. The utility of these approaches is severely limited, nonetheless, by the lack of molecular markers unique to cell types and the inability to define cellular function solely on the expression of one or more genes. Comparing single‐cell omics methods to traditional techniques that profile bulk cell populations, we can examine pathogenic and homeostatic cell populations at a resolution never before possible. The comparison between these two methods is revolutionizing our understanding of disease pathogenesis. Single‐cell analysis can study differences between cells within a cell population and may provide important clues to identify hidden disease causes in batch analysis. In addition, single‐cell multiomics approaches aim to superimpose data on DNA methylation, chromatin accessibility, proteome, and metabolome on top of gene expression patterns in individual cells. To simultaneously generate multimodal data, several experimental methods were developed that do not require physical separation of RNA and DNA from individual cells [302, 303]. These methods might be helpful in the discovery of novel treatment targets or in the creation of new fluid biomarkers of immune dysregulation, particularly when combined with machine learning‐based prediction algorithms [304]. The application of machine learning models to enable early diagnosis based on genetic and molecular data, especially multiomics data, is a particularly active area of research [304].

Single‐cell multiomics techniques have been creatively applied in developmental biology, neurology, and cancer research, particularly for assessing tumor heterogeneity and T cell infiltration [305]. Single‐cell genome, transcriptome, protein, and metabolome data may be used by single‐cell multiomics technologies to offer more in‐depth understanding of the characteristics and regulatory mechanisms of individual cells. Spatial multiomics technologies can help us understand the spatial variation of disease molecules and the molecular causes of diseases [6]. We can better understand the molecular origins of illnesses with the use of single‐cell and spatial multiomics technology. However, the application of single‐cell and spatial multiomics technologies in disease treatment is currently limited in a number of ways. Further basic research is needed to ensure the accuracy and reliability of these techniques. There are several challenges and issues that need to be resolved, including standardization and standardized data interchange methods, technical dependability, and other issues like data analysis and algorithm optimization. With the further development and improvement of related technologies, these problems will eventually be fixed and the relevant study findings will be further improved.

### 7.2 Spatial Multiomics: Mapping the Tumor Ecosystem In Situ

Future single‐cell transcriptomics research should focus on producing therapy suggestions that are both helpful to patients and helpful to physicians on a per‐patient basis. One of the most promising approaches to accomplish this aim is spatial transcriptomics, which enables direct assessment of spatially resolved gene expression in patient samples obtained from pathology. Indeed, Nature Methods named spatially resolved transcriptomics the “Method of the Year” for 2020 [306]. As technology continues to improve at a rapid pace, we expect novel methods that combine spatial transcriptomics with single‐cell multiomics to capture cellular changes with high sensitivity and resolution.

By combining several genomes methods into a unified technology that gathers data from different genomics at once, spatial multiomics makes it possible to analyze cells in tissues jointly using parallel or even identical tissue slices. It offers a three‐dimensional panoramic picture of tissues and makes it easier to analyze cell–cell interactions. Therefore, we may be able to reconstitute important carcinogenesis processes by combined profiling of spatial multiomics characteristics. Researchers have created a geographical map of human cancers and a spatial gene database, which aids in the creation of customized tumor therapy. They have also discovered spatial cellular relationships, TLS identification, and alterations in immune function using spatial multiomics technology. To help with cancer detection and therapy as well as to decipher new processes of carcinogenesis and development, it could be necessary to further develop new spatial analytic techniques and tools in the future. These tools should primarily focus on spatial and temporal resolution, throughput, and sensitivity.

### 7.3 The Promise of Multiomics Analysis of Organoids and Avatars

Organisms produced from patients are becoming more potent experimental platforms that are genetically tractable and can be integrated with single‐cell level analysis. Detecting the earliest molecular changes in cancer organoids or defining pathogenic initiation cell types could pave the way for therapies aimed at preventing pathogenic events. Therefore, it is necessary to establish a regulatory lineage for the cancer organoid cell population in order to gain a deeper understanding of the genetic risk of cancer characteristics and to predict the cell types susceptible to the disease. Additionally, organoids are thought to be a useful platform for individualized treatments in the future. It is possible to quickly use single‐cell analysis of CRC and the organoids it produced to investigate therapy approaches tailored to individual patients.

Furthermore, the use of organoids in the clinic is limited by the fact that organoid architectures are frequently diverse and nonreproductive, such as lacking cell types or having abnormal gene regulation. Understanding the molecular processes of organoid models at the genome, transcriptome and proteome levels requires multiomics investigations. An unparalleled quantitative, high‐dimensional evaluation of full molecular maps is produced by single‐cell and spatial profiling, offering reference atlases for research focused on diseases [307]. The complete picture of structures is restored on a macro‐scale by pathological and morphological analysis, which evaluates the resemblance to in vivo organs and disease/drug models in vitro. These widely used techniques accurately evaluate organoids at both the cellular and structural levels. The final piece of the puzzle about whether organoid protocols could produce a simulacrum with complex functions akin to those of genuine organ equivalents will then be completed by functional characteristics study. In fact, screening medications that fit the patient's genetic profile is especially important for aggressive cancers [308]. Therefore, quantifying biological variance in presenting original tissue in vivo and further resolving environmental perturbations on organoids in vitro models would be given greater emphasis in a future catalog of well‐assessed organoids.

### 7.4 Longitudinal Multiomics: Tracking Evolution and Therapy Resistance

There is a complicated interaction between genetic, cell state, epigenetic, spatial, and microenvironmental factors in the development of cancer. Recently, multiomics technologies have started to integrate at the crucial single‐cell level across various genetic and nongenetic factors of tumor development.

By studying human tissue, these techniques open the door to answering important questions about the development of cancer. For instance, the capacity to genotype cells for driver mutations and collect data on their transcriptional and epigenetic states is necessary to identify the variables that drive the malignant transformation versus involution of clonally grown cells. The evaluation of cancer stem cell lineage destiny decisions that result from the interplay of somatic mutations with cell states is another unexplored area of research in human malignancies. These cell states may then be influenced by extrinsic environmental signals and/or inherent epigenetic foundations, highlighting the necessity of integrating multiomics single‐cell data across modalities.

Single‐cell multiomics analysis of clonal amplification in malignant tumors and normal tissues may help to shed light on potential models for the suppression of rogue somatic evolution processes in multicellular human hosts. Recent evidence of the widespread presence of somatic driver mutations in healthy tissues raises the possibility that somatic evolution may be suppressed by genetic constraints—that is, the amount of time required for the accumulation of a large number of driver events working in tandem with other processes [309, 310]. One such mechanism could be spatial restriction, as in the case of colonic crypts, which favors drift over selection by dividing the entire population of colonic stem cells into isolated habitats, thus reducing the effective population size [311]. Therefore, increased spatial mixing over the course of malignancy may enhance selection and the emergence of treatment resistance [312]. Complex differentiation hierarchies may be another such mechanism; mathematical modeling of evolutionary dynamics supports this idea by demonstrating that evolutionary graphs reflecting differentiation hierarchies have an architecture that inhibits positive selection. It is important that these differentiation hierarchies can be transmitted either internally in spatially delimited tissues through very deep epigenetic hierarchies or extrinsically in highly ordered tissues through cytokine gradients. Clonal outgrowths often impact cytokine‐related processes in epithelial tissues and epigenetic mechanisms in hematopoietic tissue because they are a crucial initial step in overcoming this obstacle to somatic evolution. The selection of malignant clones can thus result from an efficient evolutionary process using growing clonal populations as a better substrate.

### 7.5 Building the Ecosystem: Large‐Scale Consortia, Public Databases, and Open Science

While multiomics approaches were used to discover new drug targets for the treatment of cancer, few targets were further validated by functional testing. Moreover, molecules with a specific distribution can be used not only as drug targets, but also to target drugs to selected regions. By combining materials science and spatial analysis of multiomics data, precise treatment of diseases can be elucidated by targeting certain molecular enriched cell subpopulations with drugs.

**FIGURE 4:**
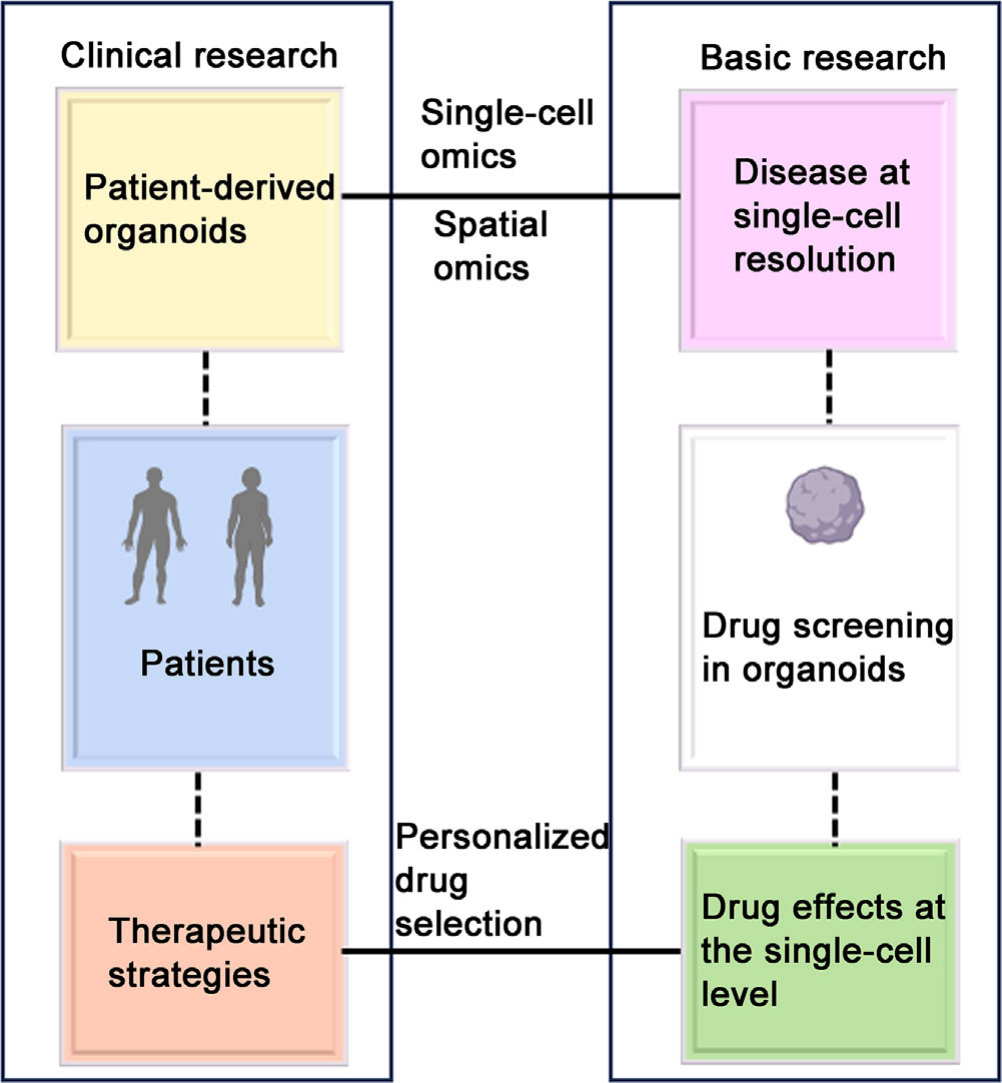
Future directions for single‐cell and spatial multiomics in clinical studies of cancer (by FigDraw). By combining single‐cell and spatial multiomics approaches with other developing technologies, it is possible to precisely comprehend cancer and create human spatial maps.

Single‐cell transcriptomics at the systemic level reveals cellular heterogeneity and biological pathways linked to oxidative stress, inflammation, and cell death that may act as molecular triggers for pathological alterations. It is essential to use cell transcriptomics in larger human cohorts and, more importantly, to create a standard method that produces similar datasets for reliable assessment of cellular phenotypes associated with disease. Currently, single‐cell techniques allow researchers to monitor molecular and cellular changes in the bloodstream, opening new avenues for disease screening as adaptive immune activation becomes more closely linked to cancer (Figure 4).

## 8 Conclusion

In cancer biology, it is crucial to identify biomarkers of cancer and targets for cancer therapy. Technological advances ushered in the era of omics, allowing the collection and integration of data across multiple molecular modalities. The data produced by omics offer novel approaches to cancer diagnosis and therapy as well as a deeper understanding of the disease pathophysiology. Single‐omics refers to investigations at the level of a single biomolecule, including genomics, transcriptomics, and proteomics. This approach sharpens the focus of the study and allows for an in‐depth exploration of biological processes and molecular mechanisms at a particular level. For example, genomics reveals genetic information about an individual, transcriptomics can provide details on gene expression, and proteomics focuses on the expression and function of proteins [313, 314, 315]. However, the limitation of single‐omics is that it ignores the interactions between different molecular levels and the complexity of regulatory networks in biological systems. Single‐omics has the drawback of ignoring the complex regulatory networks and interactions that exist at different molecular levels in biological systems. Furthermore, to fully comprehend biological systems, multiomics combines and analyzes data from several biomolecule levels, such as transcriptomics, proteomics, metabolomics, and genomes [316]. The connections and regulatory networks between many molecular levels in biological systems, as well as their dynamic changes under various situations, are studied using the multiomics approaches. This helps to reveal disease pathogenesis and search for biomarkers and therapeutic targets. Strong bioinformatics and computational biology tools and techniques are needed for multiomics, as it also confronts difficulties with data integration and interpretation. Meanwhile, the cost of collecting multiomics data is relatively high and it is necessary to appropriately reduce the cost of data collection to accelerate the application of multiomics data in healthcare services. ‌In general, single‐omics provides more depth and detail within a particular field, but multiomics provides a broader perspective and facilitates understanding of the diversity and complexity of biological systems. Future biological research will place a greater emphasis on multiomics methods as omics technologies and data processing tools develop.

As discussed above, there are a lot of potential in the nexus of omics research and machine learning thanks to the advancement of contemporary high throughput omics techniques and the resurgence of AI. Nonetheless, there are equally formidable obstacles to be surmounted. The requirement for large‐scale datasets is a crucial and fundamental component of these efforts, as next‐generation machine learning techniques, like DL, are very data‐hungry. Despite our claims to be living in the “era of big data,” many real‐world issues have small sample sizes, and DL methods cannot be applied correctly on such small datasets. Nevertheless, there are still many of ways to approach this challenge. Numerous histology data are gathered at various biological organization levels, and hundreds of variables are used to capture a wide range of biological system characteristics. While a single‐cell omics may not refer to big data, the integration of numerous omics will increase the volume of data categories. The first and foremost option is to collect and integrate multiple omics. Without advances in biotechnology, it may be prohibitively expensive to collect several omics at once. Therefore, we have to think of ways to augment or augment these data. Utilizing additional image data to characterize phenotypic changes in cell and tissue slides is an attractive alternative, since several machine learning approaches were successfully applied for diagnostic purposes. The burgeoning field of image–genomics integration is one example. Developing an imaging atlas for more cells and tissues that provide information on cellular responses to different medications or environmental perturbations might push these investigations further.

Because they are crucial to gaining a thorough knowledge of the metabolic, genetic, and molecular networks underlying cancer, multiomics approaches have garnered a great deal of interest in customizable and precision medicine. For a methodical and thorough investigation, we suggest combining several omics data to objectively define the progression of cancer. The feasibility and efficacy of multiomics approaches have led to a growing interest in this area of clinical medical research and advancements in our understanding of human cancer.

The heterogeneity of cancer is highly influenced by its etiology and pathophysiological complexity. To comprehend the pathogenesis of cancer and develop more potent therapies, research into the heterogeneity of cancer tissues is necessary. Now, we are able to sequence a single‐cell RNA or nucleus, look at the chromatin's condition, begin investigating a single cell's proteome, and even spatially resolve these data. We envision that advanced integrated technologies for measuring single‐cell transcriptomes and other patterns will allow us to study cell type characteristics in healthy and diseased humans, and will provide valuable information for new treatments for patients. We believe that developing single‐cell multiomics techniques and machine learning models for clinical use to investigate disease pathways and provide precision therapies holds great promise.

## Author Contributions

Zhenhua Du: wrote the paper draft. Xiaomei Liu, Zhi Lv, and Bengang Wang: edited the paper draft. Yu Xia and Wala Abduljabbar Mohammed Al‐Duais: reviewed the literature. Lirong Yan and Fuqiang Zhang: checked the results. Yanke Li: supervised, conceived the project, and did the funding acquisition. All data were generated in‐house, and no paper mill was used. All authors agree to be accountable for all aspects of work ensuring integrity and accuracy.

## Ethics Statement

The authors have nothing to report.

## Conflicts of Interest

None

## Data Availability

The authors have nothing to report.
